# Age-Related Differences in the Luminal and Mucosa-Associated Gut Microbiome of Broiler Chickens and Shifts Associated with *Campylobacter jejuni* Infection

**DOI:** 10.3389/fcimb.2016.00154

**Published:** 2016-11-22

**Authors:** Wageha A. Awad, Evelyne Mann, Monika Dzieciol, Claudia Hess, Stephan Schmitz-Esser, Martin Wagner, Michael Hess

**Affiliations:** ^1^Department for Farm Animals and Veterinary Public Health, Clinic for Poultry and Fish Medicine, University of Veterinary MedicineVienna, Austria; ^2^Department of Animal Hygiene, Poultry and Environment, Faculty of Veterinary Medicine, South Valley UniversityQena, Egypt; ^3^Department for Farm Animals and Veterinary Public Health, Institute of Milk Hygiene, University of Veterinary MedicineVienna, Austria

**Keywords:** broiler chickens, microbiota, 16S rRNA gene, age, luminal content, mucosa, *Campylobacter jejuni*, MiSeq sequencing

## Abstract

Despite the importance of gut microbiota for broiler performance and health little is known about the composition of this ecosystem, its development and response towards bacterial infections. Therefore, the current study was conducted to address the composition and structure of the microbial community in broiler chickens in a longitudinal study from day 1 to day 28 of age in the gut content and on the mucosa. Additionally, the consequences of a *Campylobacter (C.) jejuni* infection on the microbial community were assessed. The composition of the gut microbiota was analyzed with 16S rRNA gene targeted Illumina MiSeq sequencing. Sequencing of 130 samples yielded 51,825,306 quality-controlled sequences, which clustered into 8285 operational taxonomic units (OTUs; 0.03 distance level) representing 24 phyla. *Firmicutes, Proteobacteria, Bacteroidetes, Actinobacteria*, and *Tenericutes* were the main components of the gut microbiota, with *Proteobacteria* and *Firmicutes* being the most abundant phyla (between 95.0 and 99.7% of all sequences) at all gut sites. Microbial communities changed in an age-dependent manner. Whereas, young birds had more *Proteobacteria, Firmicutes*, and *Tenericutes* dominated in older birds (>14 days old). In addition, 28 day old birds had more diverse bacterial communities than young birds. Furthermore, numerous significant differences in microbial profiles between the mucosa and luminal content of the small and large intestine were detected, with some species being strongly associated with the mucosa whereas others remained within the luminal content of the gut. Following oral infection of 14 day old broiler chickens with 1 × 10^8^ CFU of *C. jejuni* NCTC 12744, it was found that *C. jejuni* heavily colonized throughout the small and large intestine. Moreover, *C. jejuni* colonization was associated with an alteration of the gut microbiota with infected birds having a significantly lower abundance of *Escherichia (E.) coli* at different gut sites. On the contrary, the level of *Clostridium* spp. was higher in infected birds compared with birds from the negative controls. In conclusion, the obtained results demonstrate how the bacterial microbiome composition changed within the early life of broiler chickens in the gut lumen and on the mucosal surface. Furthermore, our findings confirmed that the *Campylobacter* carrier state in chicken is characterized by multiple changes in the intestinal ecology within the host.

## Introduction

A diverse microbiota is found throughout the gastrointestinal tract (GIT) of chickens, most predominant in the cecum (Mead, 1997; Videnska et al., 2014). The gut microbiota plays an essential role in nutrition, detoxification of certain compounds, growth performance and protection against pathogenic bacteria. The microbiota is crucial to strengthen the immune system, thereby affecting growth, health, and wellbeing of chicken. Generally, the gut microbiota modulates host responses to limit the colonization of pathogens (Rehman et al., 2007). There is little information about the diversity and function of the gut microbiota in chickens, its impact on the host and the impact of certain pathogens.

Development of the gut microbiota in chickens occurs immediately after hatching and is influenced by both genetic and external factors like diet and environment (Apajalahti et al., 2004). It was reported that disturbances in the intestinal microbiota leads to a delay in growth, weakens the host resistance and increases the susceptibility to various infectious diseases (Lan et al., 2004). Gong et al. (2002) demonstrated that the cecal microbiota protects chickens against bacterial infections, while microbiota in the small intestine contributes significantly to its function, including digestion and nutrient absorption, which significantly determines the growth rate of the bird. Studies on gut microbiota have mostly been performed with chickens older than 1 week of age due to the various influences in day-old birds. However, the composition of gut microbiota at the first day of life in newly hatched chickens is a matter of interest within a longitudinal study. Therefore, the focus of the actual study was to determine the diversity and community structures of the microbiota within the small and large intestine from hatch until 4 weeks of age. Furthermore, differences among the mucosa-associated and luminal content microbiota were determined for the first time.

*Campylobacter (C.) jejuni* is the most common cause of food-borne bacterial enteritis worldwide (EFSA, 2011). *C. jejuni* infection of chickens had previously not been considered to influence bird health and it was thought that *C. jejuni* is part of the normal microbiota of birds (EFSA, 2011). Understanding how *Campylobacter* species, especially *C. jejuni*, establishes successful colonization in chickens remains a foremost research priority as this gastrointestinal pathogen not only overcomes the host's defense system, but also competes with the microbial community for space and nutrients.

It has been shown that *Campylobacter* requires numerous factors to successfully colonize the host, to translocate and to avoid clearance (Awad et al., 2014, 2015a,b, 2016; Humphrey et al., 2014). In addition, Awad et al. (2016) showed that *Campylobacter* had the ability to reduce butyrate, isobutyrate, valerate, and isovalerate which might be due to the utilization of short-chain fatty acids (SCFAs) as a carbon source (Masanta et al., 2013) or due to the reduction of butyric acid producing bacteria amongst the microbiota. In general, there is a complex interplay between microbiota composition and SCFAs concentration and it was found that the type and level of SCFAs in the gut can affect different members of the microbial community in various ways (Mon et al., 2015).

It is still unknown how *C. jejuni* affects the ecology of the chicken gut, a feature of high importance considering a possible detrimental effect on the health of birds associated with *C. jejuni* colonization. Haag et al. (2012) demonstrated that *C. jejuni* colonization in mice depends on the microbiota of the host and *vice versa* and *Campylobacter* colonization induces a shift of the intestinal microbiota. Thus, it can be hypothesized that *Campylobacter* colonization is associated with an alteration in the intestinal microbiota of chickens as well. Therefore, the second aim of the actual study was to investigate the dynamics of an experimental *Campylobacter jejuni* NCTC 12744 infection in 14–28 days old chickens and the consequences on the alteration of the gut microbiome.

## Materials and methods

### Ethics statement

The animal experiment was approved by the institutional ethics committee of the University of Veterinary Medicine and the Ministry of Research and Science under the license number GZ 68.205/0011-11/3b/2013. All husbandry practices were performed with full consideration of animal welfare.

### Experimental design

In this study, a total of 45 1-day-old broiler chickens (males and females) were obtained from a commercial hatchery (Ross-308, Geflügelhof Schulz, Graz, Austria). Five day-old birds were immediately sacrificed for determining the gut microbiota of the jejunal and cecal mucosa. At 7 and 14 days of age, five birds were randomly selected for measuring the development of gut microbiota from gut content and mucosa. The birds were kept as non-infected for the first 2 weeks and were housed on wood shavings with feed and water supplied *ad libitum*. The birds were fed a standard commercial diet for the whole experimental period in order to avoid an influence of the change of diet on the microbial composition.

At the first and 14 days of age birds were confirmed as *Campylobacter*-free by taking cloacal swabs which were streaked onto modified charcoal-cefaprazone-deoxycholate agar (CM0739, OXOID, Hampshire, UK) and grown for 48 h under microaerophilic conditions at 42°C. At 14 days of age, 15 birds were infected with *Campylobacter jejuni (C. jejuni)* reference strain NCTC 12744 and kept separately from 15 non-infected control birds which were inoculated with PBS only. *C. jejuni* was routinely grown in Lennox L Base broth (LB broth) (Invitrogen, California, USA) at 42°C for 48 h in a shaking incubator. *Campylobacter* colony-forming unit (CFU) was determined from each suspension by serial dilutions in duplicate using LB agar. *Campylobacter* suspensions were stored at −80°C by adding 2 mL of 40% glycerol/10 mL LB broth. For infection, *Campylobacter* suspensions were centrifuged for 5 min at 10,000 × rpm. The pellet was washed 3 times with phosphate-buffered saline (PBS) each time centrifuged at the same conditions as mentioned above. Finally, the pellets were resuspended in PBS and the necessary concentration was adjusted for birds' infection.

The infection was performed orally via feeding tube (gavage) with a dose of 1 × 10^8^ CFU/bird at 14 days of age as described previously (Awad et al., 2015a). At 7 days post infection 5 birds from each group were anesthetized by injection of a single dose of thiopental (20 mg/kg) into the wing vein and slaughtered by bleeding of the jugular vein. The final 10 birds/group were killed at 14 days post infection. At each time point the gastrointestinal content from the jejunum and ceca, as well as jejunal and cecal mucosa from 5 birds/group were taken to determine the gut microbiota. Intestinal segments were disclosed at the mesentery with sterile instruments and the digesta was removed. The luminal site of the intestinal segments was washed with sterile ice-cold PBS until the mucosa was completely cleaned from the digesta. The mucosa was rinsed several times with sterile ice cold PBS, after which the mucosa was collected aseptically by scraping off the mucosa using scalpel blades. All samples were stored at −80°C until further processing.

### DNA extraction, PCR amplification of the 16S rRNA gene, and illumina MISeq sequencing

DNA from luminal content and gut mucosa samples was extracted using the PowerSoil® DNA Isolation Kit (MoBio Laboratories, Carlsbad, CA, USA) as described previously (Mann et al., 2014; Yasuda et al., 2015). The same protocol of DNA extraction was applied to luminal content and gut mucosa. From each of the 130 samples a total of 250 mg of gut content or mucosa was used for DNA isolation according to manufacturer's instructions. DNA concentration was determined by a Qubit fluorometer (Invitrogen, Carlsbad, CA, USA). The V345 hypervariable region of the 16S rRNA genes was amplified with the primers F341 (5′-GTGYCAGCMGCCGCGGTAA-3′) (Zakrzewski et al., 2012) and R909 (5′-CCGYCAATTYMTTTRAGTTT-3′) (Tamaki et al., 2011). An amplicon size of approximately 568 bp was produced.

16S rRNA gene PCRs, library preparation and sequencing were performed by Microsynth (Microsynth AG, Balgach Switzerland). Libraries were constructed by ligating sequencing adapters and indices onto purified PCR products using the Nextera XT Sample Preparation Kit (Illumina) and equimolar amounts of each of the libraries were pooled and sequenced on an Illumina MiSeq Sequencing Platform. Sequence data were analyzed with the software package QIIME (Caporaso et al., 2010). Low quality sequences (*q* < 20) were filtered, chimeric sequences were excluded by using the USEARCH 6.1 database (Edgar, 2010) and sequences were clustered into operational taxonomic units (OTUs; 97% similarity) with the QIIME script “pick_open_reference_otus.py.” OTUs with less than 10 sequences were removed, resulting in 8285 OTUs, which were used for all downstream analysis. The representative sequences of the 50 most abundant OTUs over all sampling time points were classified against type strains using the Greengenes database (http://greengenes.lbl.gov) (DeSantis et al., 2006).

### Microbial diversity analysis

Both alpha and beta diversity indices were used to estimate the microbiome diversity within—and between microbial communities. Calculations were done with the “summary.single” command in the software package mothur (http://www.mothur.org/; Kozich et al., 2013). Alpha diversity indices analysis included Chao1 index (richness estimate), abundance-based coverage estimator (ACE, richness estimate), Shannon's diversity, and Simpson's diversity index.

For the Bray-Curtis similarity, the dataset was rarefied to the minimum number of sequences per sample. Rarefaction curve was constructed based on the observed number of OTUs and nearly reached asymptotes for all samples (data not shown).

Principal component analysis was done with JMP® (Version 10.0.0, SAS Institute Inc., Cary, NC). Shared OTUs among gut sites at different age were plotted as Venn diagrams using the R environment (package “VennDiagram,” version 1.6.17.) (Chen, 2016). Heatmaps were created using JColorGrid (Joachimiak et al., 2006).

### Statistical analysis

Statistically significant differences in relative abundance with regard to sampling sites and time were calculated using “metastats” in mothur, which is based on the homonymous bioinformatics program (White et al., 2009; Paulson et al., 2011). “Metastats” uses repeated *t* statistics and Fisher's tests on random permutations to handle sparsely-sampled features (White et al., 2009). Results were reported as a mean and standard deviation (*SD*). The significance level was set to *P* < 0.05. The *P*-values were adjusted with the Benjamini and Hochberg false discovery rate correction (FDR, *q*-value), and a *q* < 0.25 was considered significant (Lim et al., 2016). Furthermore, significant differences between the diversity estimators of the two groups were performed using the non-parametric Kruskal-Wallis-test followed by Mann-Whitney-test. PASW statistics 20, SPSS software (Chicago, Il., USA) was used for statistical analyses of diversity estimators.

### Accession numbers

Sequencing data are available in the European Nucleotide Archive (ENA) database under the accession number PRJEB14860.

## Results

### Sequence analysis, phylum and OTU classification

Sequencing of 130 samples yielded 51,825,306 quality-controlled sequences, clustering into 8285 operational taxonomic units (OTUs; 0.03 distance level). Throughout all gut sites 24 phyla were identified with *Firmicutes, Proteobacteria*, and *Tenericutes* being the most abundant ones. In Figure S1A, Tables S1A–D, relative abundances of all phyla are delineated with respect to age and groups. The results showed that in the jejunum and cecum, *Firmicutes* and *Proteobacteria* were the dominating luminal and mucosal-associated phyla in all birds investigated (Tables S2, S3).

At the first day of life *Proteobacteria* were significantly higher in the jejunal (*P* = 0.0000, *q* = 0.0000) and cecal (*P* = 0.016; *q* = 0.059) mucosa of the birds and decreased thereafter, as no significant differences were found between samples from day 14 to day 28 of age (*P* = 0.140; *q* = 0.438 and *P* = 0.519; *q* = 0.955). On the contrary, *Firmicutes* were significantly lower at day 1 and increased thereafter (*P* = 0.001; *q* = 0.016 and *P* = 0.006; *q* = 0.055 in the jejunal and cecal mucosa, respectively).

**Figure 1:**
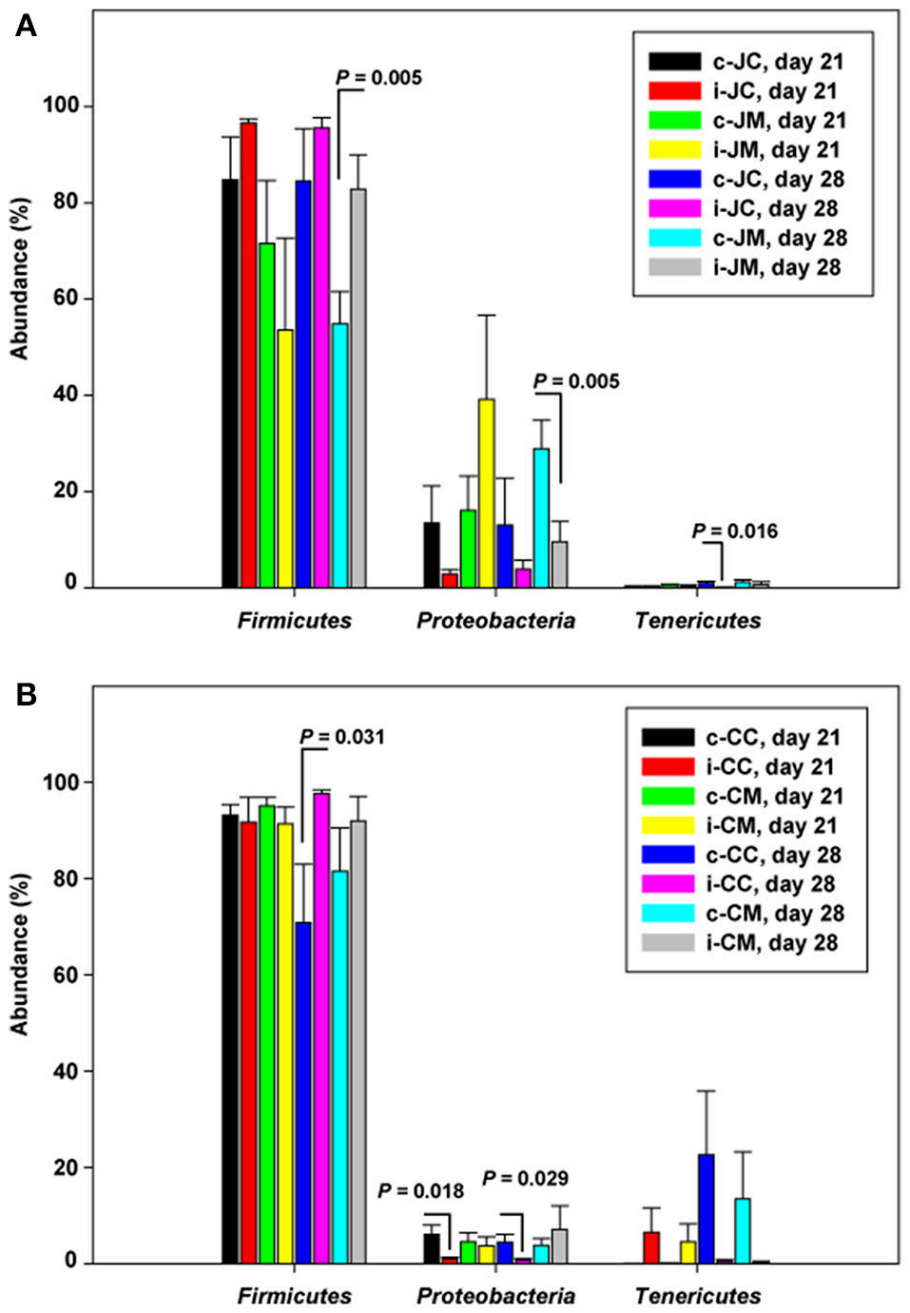
**Relative abundances (%) of the most abundant phyla in the infected birds compared with the controls at the two sampling points post infection of (A)** jejunum and **(B)** cecum. Data are presented as the mean values and standard deviation (*SD*). JM, jejunal mucosa; JC, jejunal content; CM, cecum mucosa; CC, cecum content; control (c); infected (i).

For infected birds, relative abundances of bacterial phyla at the two sampling time points carried out post infection are represented in Figure S1B, Tables S4A–D. Figure 1 shows that the phylum *Proteobacteria* decreased while *Firmicutes* increased at either 21 (7 dpi) or 28 days of age (14 dpi). There was a significant decrease in *Actinobacteria* and *Proteobacteria* in the jejunal mucosa at 14 dpi (*P* = 0.006; *q* = 0.100 and *P* = 0.005; *q* = 0.100), while *Firmicutes* and *Bacteroidetes* were more abundant in the infected birds compared to the controls (*P* = 0.005; *q* = 0.100 and *P* = 0.023; *q* = 0.217, Table S4A). However, in the cecal content and cecal mucosa, *Bacteroidetes* (*P* = 0.001; *q* = 0.019) increased at 7 dpi, but decreased (*P* = 0.002; *q* = 0.026 and *P* = 0.005; *q* = 0.048) at 14 dpi in the infected birds compared with the controls, indicating that the *Campylobacter* infection modulates the jejunal and cecal phylum abundances in different ways.

In Table 1, the 50 most abundant OTUs from all birds are listed including the internal OTU number, relative abundance together with the reference strain and similarity (compared with strains of the Greengenes database). Relative OTUs abundances at different ages in all birds are shown in Tables S5A–D, S6A–D. The OTUs and species abundances sorted by age at the four gut sites of the birds are shown in the heatmaps of Figure S2. In total, the 50 most abundant OTUs accounted for 73.9% of all sequences and of those 42 OTUs differed significantly in their relative abundances over all gut sites independent of the age (Tables 2, 3). At the first day of age, a notable high relative abundance of OTU 1, 25, 27, and 35 (best type strain hits: *Escherichia coli, Enterococcus faecalis, Clostridium paraputrificum*, and *Clostridium sartagoforme*) were found in both jejunal and cecal mucosa (Tables S5A,C), whereas OTU 38 (best type strain hit: *Acinetobacter johnsonii*) was only abundant in the jejunal mucosa and OTU 42 (best type strain hit: *C*. *paraputrificum*) was only abundant in the cecal mucosa. All these abundant OTUs decreased by age. In the jejunal mucosa, OTU 1 was the most abundant (57.9%), followed by the other four OTUs which ranged between 2.6 and 7.9%. Similarly, in the mucosa of the cecum, OTU 1 was highly abundant (65.9%), followed by OTUs 27, 25, 35, and 42 which ranged between 7.8 and 3.3%.

**Table 1:** **The 50 most abundant OTUs retrieved from different gut sites from all birds independent of the infection status**.

**OTUs**	**No. of sequences**	**Relative abundance (%)**	**Best type strain hit^a^**	**Similarity (%)**
OTU 1	642068	10.64	*Escherichia coli* (GU968184.1)	100
OTU 2	341923	5.66	*Lactobacillus johnsonii* (HM772969.1)	100
OTU 3	339288	5.62	*Lactobacillus salivarius* (NZ_AEBA01000145.1)	100
OTU 4	310060	5.14	*Clostridium* spp. (FJ808599.1)	87
OTU 5	273783	4.54	*Enterococcus durans* (FJ917726.1)	100
OTU 6	209121	3.46	*Anaerotruncus colihominis* (NR_027558.1)	100
OTU 7	208070	3.45	*Eubacterium desmolans* (L34618.1)	98
OTU 8	161864	2.68	*Clostridium cellulolyticum* (X71847.1)	87
OTU 9	157466	2.61	*Spiroplasma lampyridicola* (AY189134.1)	82
OTU 10	151864	2.52	*Clostridium leptum* (AJ305238.1)	97
OTU 11	130587	2.16	*Eubacterium desmolans* (L34618.1)	99
OTU 12	127446	2.11	*Eubacterium desmolans* (L34618.1)	98
OTU 13	120732	2.00	*Clostridium papyrosolvens* (NR_026102.1)	87
OTU 14	113436	1.88	*Eubacterium coprostanoligenes* (HM037995.1)	92
OTU 15	98491	1.63	*Clostridium straminisolvens* (NR_024829.1); *Clostridium thermocellum* (AB558166.1)	87
OTU 16	87863	1.46	*Clostridium cellulolyticum* (NC_011898.1)	87
OTU 17	70842	1.17	*Bacillus subtilis* (FJ608705.1)	94
OTU 18	67038	1.11	*Lactobacillus reuteri* (EU547311.1)	99
OTU 19	66212	1.10	*Ruminococcus bromii* (NR_025930.1)	94
OTU 20	65592	1.09	*Eubacterium desmolans* (L34618.1)	95
OTU 21	53133	0.88	*Clostridium cellulolyticum* (NC_011898.1); *Clostridium papyrosolvens* (NR_026102.1)	87
OTU 22	49736	0.82	*Clostridium thermocellum* (AB558166.1)	85
OTU 23	47570	0.79	*Clostridium thermosuccinogenes* (Y18180.1); *Clostridium thermocellum* (AB558166.1)	86
OTU 24	40373	0.67	*Clostridium leptum* (AJ305238.1)	95
OTU 25	36925	0.61	*Enterococcus faecalis* (FJ607291.1)	100
OTU 26	35311	0.58	*Proteus mirabilis* (GU477712.1)	100
OTU 27	31154	0.52	*Clostridium paraputrificum* (AY442815.1)	100
OTU 28	29186	0.48	*Variovorax paradoxus* (HQ005421.1)	100
OTU 29	28876	0.48	*Campylobacter* subsp. *jejuni* (NC_008787.1)	100
OTU 30	27988	0.46	*Clostridium leptum* (AJ305238.1)	93
OTU 31	23587	0.39	*Anaerotruncus colihominis* (NR_027558.1)	93
OTU 32	23559	0.39	*Lactobacillus crispatus* (FN692037.1)	100
OTU 33	22863	0.38	*Phyllobacterium myrsinacearum* (AY785330.1)	100
OTU 34	21547	0.36	*Catabacter hongkongensis* (AY574991.1); *Clostridium thermosuccinogenes* (Y18180.1)	86
OTU 35	20378	0.34	*Clostridium sartagoforme* (FJ384380.1)	100
OTU 36	18849	0.31	*Acetivibrio cellulolyticus* (L35515.1)	95
OTU 37	18055	0.30	*Clostridium papyrosolvens* (NR_026102.1)	86
OTU 38	17890	0.30	*Acinetobacter johnsonii* (EU977694.1)	100
OTU 39	15658	0.26	*Clostridium papyrosolvens* (NR_026102.1)	87
OTU 40	15361	0.25	*Lactobacillus johnsonii* (EU381128.1)	96
OTU 41	15143	0.25	*Clostridium thermosuccinogenes* (Y18180.1)	87
OTU 42	14985	0.25	*Clostridium paraputrificum* (AY442815.1)	100
OTU 43	14682	0.24	*Marinobacter* sp. (FJ889664.1)	99
OTU 44	14652	0.24	*Clostridium papyrosolvens* (NR_026102.1)	87
OTU 45	14304	0.24	*Shigella flexneri* (CP000266.1)	99
OTU 46	13619	0.23	*Clostridium cellulolyticum* (NC_011898.1)	86
OTU 47	13307	0.22	*Clostridium papyrosolvens* (NR_026102.1)	86
OTU 48	13152	0.22	*Clostridium papyrosolvens* (NR_026102.1)	87
OTU 49	12978	0.21	*Clostridium papyrosolvens* (NR_026102.1)	87
OTU 50	12418	0.21	*Lactobacillus salivarius* (NZ_AEBA01000145.1)	98

a *Greengenes best type strain hit accession numbers are listed in parenthesis*.

**Table 2:** **Relative abundances (%) of the most abundant OTUs in different gut sites of control birds (day 1–28)**.

**OTUs ID**	**Jejunum mucosa**		**Jejunum content**		**Cecum mucosa**		**Cecum content**		**JM-JC**		**CM-CC**		**JM-CM**		**JC-CC**	
	**Mean**	***SD***	**Mean**	***SD***	**Mean**	***SD***	**Mean**	***SD***	***P*-values**	***q*-values^a^**	***P*-values**	***q*-values^a^**	***P*-values**	***q*-values^a^**	***P*-values**	***q*-values^a^**
OTU 49	0	0.02	0.05	0.11	0.01	0.02	0.01	0.02	**0.056**	**0.164**	0.655	0.858	0.366	0.623	0.133	0.326
OTU 25	1.89	4.01	0.06	0.07	1.05	2.95	0.01	0.02	**0.001**	**0.011**	**0.015**	**0.067**	0.365	0.623	**0.001**	**0.011**
OTU 5	12.41	25.84	13.09	21.85	0.28	0.56	0.07	0.09	0.920	0.990	**0.039**	**0.134**	**0.002**	**0.017**	**0.001**	**0.011**
OTU 30	0.45	1.45	0.40	0.72	0.74	1.11	1.25	2.09	0.913	0.990	0.385	0.643	0.808	0.946	**0.059**	**0.170**
OTU 9	0.90	2.72	1.03	3.05	3.56	9.78	5.82	15.04	0.938	0.990	0.514	0.765	0.196	0.401	**0.091**	0.244
OTU 13	1.34	5.22	0.75	1.84	1.36	4.51	1.41	4.43	0.714	0.890	0.831	0.946	0.940	0.990	0.549	0.770
OTU 15	0.07	0.20	0.13	0.32	1.17	1.98	4.21	9.38	0.792	0.942	**0.095**	**0.250**	**0.005**	**0.030**	**0.001**	**0.011**
OTU 18	2.36	8.46	2.34	6.03	0.08	0.23	0.03	0.09	0.961	0.994	0.409	0.665	0.081	0.220	**0.013**	**0.059**
OTU 21	0.65	2.01	0.32	0.87	0.53	1.11	1.08	3.21	0.588	0.787	0.599	0.796	0.812	0.946	0.294	0.526
OTU 33	0.96	1.38	2.57	5.92	0	0	0	0	0.341	0.603			**0.001**	**0.011**		
OTU 12	0.95	2.20	1.25	4.71	2.42	5.21	5.41	11.82	0.776	0.929	0.285	0.519	0.491	0.739	0.131	0.326
OTU 28	3.21	7.19	2.99	7.77	0	0	0	0	0.941	0.990	**0.006**	**0.034**	**0.001**	**0.011**	**0.001**	**0.011**
OTU 37	0.12	0.56	0	0.01	0.39	1.93	0.07	0.28	0.140	0.326	0.939	0.990	0.947	0.991	0.177	0.379
OTU 40	0.05	0.11	1.08	1.53	0	0	0.10	0.44	**0.001**	**0.011**	0.464	0.722	**0.001**	**0.011**	**0.008**	**0.043**
OTU 3	10.36	20.66	18.43	23.28	0.39	0.63	1.52	5.30	0.252	0.475	0.517	0.765	**0.001**	**0.011**	**0.002**	**0.017**
OTU 26	0.03	0.07	0.03	0.06	1.34	2.35	1.75	3.25	0.483	0.737	0.691	0.875	**0.001**	**0.011**	**0.003**	**0.020**
OTU 19	0.80	1.99	0.22	0.57	1.09	2.22	1.01	2.17	0.189	0.395	0.979	0.997	0.961	0.994	0.134	0.326
OTU 44	0.04	0.15	0.02	0.04	0.06	0.12	0.07	0.14	0.748	0.908	0.660	0.858	0.452	0.715	**0.052**	**0.155**
OTU 39	0.02	0.07	0.01	0.02	0.07	0.20	0.08	0.19	0.769	0.927	0.726	0.893	0.253	0.475	**0.046**	**0.147**
OTU 29	0	0	0	0	0	0	0	0			**0.002**	**0.017**				
OTU 2	10.86	14.76	24.38	24.19	0.13	0.26	0.31	1.13	**0.046**	**0.147**	0.817	0.946	**0.001**	**0.011**	**0.001**	**0.011**
OTU 38	1.51	5.31	0.17	0.67	0	0	0	0	0.145	0.326			**0.002**	**0.017**		
OTU 42	0.51	1.20	0.01	0.03	0.67	1.71	0.01	0.03	**0.012**	**0.057**	**0.041**	**0.136**	0.717	0.890	0.669	0.864
OTU 17	0.46	1.30	0.02	0.05	2.79	7.57	0.05	0.15	**0.041**	**0.136**	**0.063**	**0.176**	0.243	0.466	0.571	0.770
OTU 1	14.87	24.08	5.33	7.93	22.17	26.59	13.03	17.69	**0.069**	**0.190**	0.231	0.452	0.295	0.526	**0.062**	**0.176**
OTU 11	0.69	1.32	0.51	0.97	2.63	7.62	3.10	5.11	0.362	0.623	0.692	0.875	0.458	0.719	**0.003**	**0.020**
OTU 4	0.51	2.00	0.17	0.56	1.34	4.60	2.14	7.58	0.571	0.770	0.611	0.806	0.525	0.770	0.147	0.326
OTU 48	0	0	0	0	0	0	0	0			**0.036**	**0.131**				
OTU 14	0.24	0.69	0.22	0.43	3.77	8.30	4.66	10.97	0.542	0.770	0.747	0.908	**0.023**	**0.091**	**0.004**	**0.025**
OTU 46	0	0.02	0.01	0.02	0.26	0.65	0.25	0.50	0.348	0.609	0.991	1.000	**0.028**	**0.106**	**0.039**	**0.134**
OTU 31	0.23	0.55	0.05	0.12	0.87	1.67	0.33	0.51	0.137	0.326	0.170	0.368	0.166	0.364	**0.016**	**0.068**
OTU 45	0.05	0.09	0.01	0.01	0.35	0.56	1.02	1.62	**0.023**	**0.091**	**0.096**	**0.250**	**0.016**	**0.068**	**0.004**	**0.025**
OTU 10	0.37	1.03	0.20	0.46	4.39	11.22	4.97	10.40	0.477	0.736	0.824	0.946	**0.013**	**0.059**	**0.003**	**0.020**
OTU 32	0.12	0.43	0.23	0.79	0	0	0	0	0.540	0.770	0.868	0.975	**0.012**	**0.057**	**0.006**	**0.034**
OTU 8	0.28	1.29	0.03	0.10	1.43	5.01	2.34	7.10	0.537	0.770	0.551	0.770	0.370	0.624	0.138	0.326
OTU 6	1.17	3.54	0.13	0.28	9.20	15.81	1.39	1.56	0.238	0.461	**0.007**	**0.039**	**0.021**	**0.087**	**0.001**	**0.011**
OTU 36	0.03	0.11	0.11	0.22	0.68	1.66	0.25	0.49	0.144	0.326	0.263	0.488	**0.003**	**0.020**	0.220	0.445
OTU 35	0.57	1.28	0.02	0.04	0.90	2.13	0.02	0.08	**0.012**	**0.057**	**0.034**	**0.126**	0.542	0.770	0.867	0.975
OTU 7	1.07	2.73	1.41	2.66	3.52	7.28	6.47	12.19	0.832	0.946	0.282	0.518	0.125	0.320	**0.037**	**0.132**
OTU 16	0.73	3.50	0.27	1.01	1.41	3.77	1.96	4.92	0.905	0.990	0.562	0.770	0.400	0.656	0.146	0.326
OTU 27	0.98	2.31	0.02	0.07	1.56	4.02	0.01	0.05	**0.012**	**0.057**	**0.048**	**0.151**	0.563	0.770	0.719	0.890
OTU 20	1.68	4.52	0.05	0.18	0.86	4.28	0.91	4.07	**0.049**	**0.151**	0.981	0.997	0.448	0.715	0.997	1.000
OTU 50	0.09	0.15	0.84	0.95	0	0.01	0.06	0.23	**0.001**	**0.011**	0.486	0.737	**0.001**	**0.011**	**0.002**	**0.017**
OTU 22	0	0	0	0.02	0	0	0	0.01			0.231	0.452			0.882	0.985
OTU 47	0.12	0.53	0	0.02	0.32	1.55	0.06	0.22	0.191	0.395	0.917	0.990	0.933	0.990	0.180	0.381
OTU 41	0	0.01	0	0	0	0	0	0			0.228	0.452	1.000	1.000		
OTU 23	0.12	0.50	0.01	0.02	0.07	0.20	0.09	0.23	0.136	0.326	0.682	0.874	0.819	0.946	**0.024**	**0.093**
OTU 43	0.01	0.03	0	0	0	0	0	0								
OTU 34	0.03	0.14	0	0	0.06	0.16	0.14	0.38	0.903	0.990	0.417	0.672	0.390	0.645	**0.051**	**0.155**
OTU 24	0.11	0.52	0.18	0.39	1.18	1.89	1.10	1.90	0.567	0.770	0.973	0.997	**0.001**	**0.011**	**0.003**	**0.020**

a *q-value: The False Discovery Rate (FDR) adjusted p-value using Benjamini and Hochberg method and the q < 0.25 after FDR correction considered significant. Statistically significant values are formatted in bold*.

**Table 3:** **Relative abundances (%) of the most abundant OTUs in different gut sites of infected birds (days 21 and 28)**.

**OTUs ID**	**Jejunum mucosa**		**Jejunum content**		**Cecum mucosa**		**Cecum content**		**JM-JC**		**CM-CC**		**JM-CM**		**JC-CC**	
	**Mean**	***SD***	**Mean**	***SD***	**Mean**	***SD***	**Mean**	***SD***	***P*-values**	***q*-values^a^**	***P*-values**	***q*-values^a^**	***P*-values**	***q*-values^a^**	***P*-values**	***q*-values^a^**
OTU 49	0.18	0.28	0.43	0.98	0.61	1.44	0.52	0.91	0.689	0.880	0.936	0.989	0.550	0.791	0.516	0.776
OTU 25	0.02	0.04	0.08	0.09	0.00	0.00	0	0	**0.077**	0.178			**0.063**	0.168		
OTU 5	1.65	2.21	8.89	6.18	0.01	0.01	0	0	**0.009**	**0.043**	0.761	0.927	**0.001**	**0.007**	**0.001**	**0.007**
OTU 30	0.31	0.94	0.06	0.08	0.30	0.38	0.16	0.12	0.957	0.989	0.355	0.596	1.000	1.000	**0.013**	**0.056**
OTU 9	0.17	0.39	0.14	0.14	1.47	4.29	2.06	5.88	0.903	0.987	0.575	0.791	0.530	0.785	0.217	0.408
OTU 13	0.64	1.06	0.72	1.35	2.87	3.77	2.65	2.58	0.955	0.989	0.950	0.989	**0.056**	0.159	**0.020**	**0.071**
OTU 15	0.08	0.13	0.01	0.02	1.24	0.89	2.13	1.23	**0.049**	0.146	**0.040**	0.126	**0.001**	**0.007**	**0.001**	**0.007**
OTU 18	4.67	9.39	6.34	11.77	0.19	0.44	0.14	0.36	0.572	0.791	0.857	0.986	**0.102**	**0.218**	**0.011**	**0.051**
OTU 21	0.28	0.58	0.16	0.19	1.63	2.60	1.93	2.41	0.565	0.791	0.611	0.611	**0.085**	**0.191**	**0.009**	**0.043**
OTU 33	1.11	1.14	0.13	0.13	0.00	0.00	0.00	0.00	**0.008**	**0.041**	1.000	1.000	**0.001**	**0.007**	**0.001**	**0.007**
OTU 12	0.51	0.74	0.06	0.08	0.06	0.09	0.05	0.05	**0.056**	**0.159**	0.929	0.989	**0.062**	**0.168**	0.796	0.949
OTU 28	1.19	1.20	0.20	0.21	0.00	0.00	0.00	0.00	**0.015**	**0.060**	**0.016**	**0.060**	**0.001**	**0.007**	**0.001**	**0.007**
OTU 37	0.14	0.27	0.01	0.02	0.35	0.18	0.51	0.27	**0.074**	**0.178**	**0.077**	**0.178**	0.207	0.394	**0.001**	**0.007**
OTU 40	0.07	0.08	1.35	0.83	0.01	0.01	0.00	0.01	**0.001**	**0.007**	0.881	0.987	**0.008**	**0.041**	**0.001**	**0.007**
OTU 3	11.02	13.33	29.61	19.69	0.52	0.56	0.41	0.45	**0.067**	**0.173**	0.865	0.986	**0.004**	**0.023**	**0.001**	**0.007**
OTU 26	0	0	0.01	0.05	0.01	0.01	0.01	0.01	0.542	0.791	0.801	0.949	**0.070**	**0.176**	0.990	1.000
OTU 19	0.14	0.29	0.31	0.24	3.24	7.48	2.09	5.77	0.372	0.613	0.614	0.823	**0.107**	**0.223**	0.441	0.700
OTU 44	0.43	1.20	0.07	0.12	0.49	0.94	1.06	2.17	0.594	0.809	0.574	0.791	0.552	0.791	**0.014**	**0.059**
OTU 39	0.02	0.05	0.13	0.20	0.59	0.79	1.02	1.34	**0.072**	**0.178**	0.318	0.539	**0.001**	**0.007**	**0.016**	**0.060**
OTU 29	0.30	0.66	0.01	0.03	3.92	6.25	0.38	0.51	**0.052**	**0.152**	**0.049**	**0.146**	**0.036**	**0.115**	**0.005**	**0.028**
OTU 2	19.98	22.32	23.46	10.10	0.85	1.46	0.19	0.30	0.834	0.975	0.285	0.493	**0.002**	**0.012**	**0.001**	**0.007**
OTU 38	0	0.01	0	0	0	0	0	0	0.690	**0.012**						
OTU 42	0	0	0	0	0	0	0	0								
OTU 17	0.62	1.33	0.25	0.31	4.45	6.58	0.12	0.18	0.451	0.703	**0.002**	**0.012**	**0.091**	**0.201**	0.531	0.785
OTU 1	0.11	0.18	0.81	1.36	0.28	0.40	0.19	0.36	0.145	0.292	0.760	0.927	0.280	0.491	0.255	0.455
OTU 11	1.09	1.39	0.10	0.10	2.01	2.44	2.31	3.56	**0.026**	**0.086**	0.761	0.927	0.907	0.987	**0.007**	**0.038**
OTU 4	3.43	6.60	0.13	0.11	12.51	8.03	16.82	10.92	0.154	0.303	0.162	0.315	**0.016**	**0.060**	**0.001**	**0.007**
OTU 48	0.41	1.14	0.01	0.02	2.29	7.23	0.38	1.17	0.225	0.414	0.747	0.927	0.817	0.962	0.222	0.413
OTU 14	0.24	0.65	0.07	0.07	0.45	0.84	1.41	2.08	0.364	0.606	0.122	0.251	0.619	0.824	**0.076**	**0.178**
OTU 46	0.21	0.60	0.01	0.02	0.49	0.81	0.52	0.87	0.306	0.524	0.757	0.927	0.396	0.646	**0.016**	**0.060**
OTU 31	0.03	0.06	0.03	0.04	0.37	0.52	0.08	0.07	0.955	0.989	**0.057**	**0.159**	**0.012**	**0.055**	**0.019**	**0.068**
OTU 45	0	0	0	0	0	0	0	0			1.000	1.000			0.281	0.491
OTU 10	0.26	0.82	0.09	0.12	0.29	0.57	0.22	0.30	0.960	0.989	0.896	0.987	0.891	0.987	0.175	0.336
OTU 32	2.90	3.72	3.80	3.98	0.18	0.46	0.05	0.11	0.872	0.986	0.567	0.791	**0.004**	**0.023**	**0.001**	**0.007**
OTU 8	1.21	2.88	0.05	0.09	7.75	7.99	6.25	8.70	**0.036**	**0.115**	0.917	0.989	**0.021**	**0.073**	**0.001**	**0.007**
OTU 6	0.82	1.99	0.10	0.13	4.11	2.46	1.24	1.97	0.455	0.703	**0.026**	**0.086**	**0.019**	**0.068**	**0.001**	**0.007**
OTU 36	0.21	0.40	0.23	0.21	0.34	0.37	0.19	0.22	0.893	0.987	0.441	0.700	0.473	0.724	0.871	0.986
OTU 35	0	0	0	0	0	0	0	0								
OTU 7	0.24	0.40	0.08	0.17	4.65	13.62	6.23	18.65	**0.095**	**0.205**	0.692	0.880	0.501	0.760	**0.062**	**0.168**
OTU 16	0.19	0.27	0.29	0.47	1.37	1.80	1.55	1.94	0.776	0.932	0.653	0.862	**0.074**	**0.178**	**0.048**	**0.146**
OTU 27	0	0	0	0	0	0	0	0								
OTU 20	0.22	0.67	0.03	0.05	4.32	13.62	0.62	1.88	0.849	0.986	0.770	0.932	0.576	0.791	0.424	0.686
OTU 50	0.10	0.17	1.20	0.73	0.01	0.01	0.01	0.01	**0.001**	**0.007**	0.979	1.000	**0.013**	**0.056**	**0.001**	**0.007**
OTU 22	0.14	0.42	0	0	2.08	5.32	3.60	9.82	**0.107**	**0.223**	0.671	0.877	0.236	0.430	**0.001**	**0.007**
OTU 47	0.03	0.06	0.01	0.01	0.20	0.10	0.31	0.23	0.244	0.440	**0.092**	**0.201**	**0.001**	**0.007**	**0.001**	**0.007**
OTU 41	0.99	2.99	0	0	0.50	0.87	1.28	3.32			0.723	0.913	0.923	0.989		
OTU 23	0.63	1.18	0.02	0.02	2.39	2.31	2.88	2.65	0.124	0.252	0.448	0.703	**0.064**	**0.168**	**0.001**	**0.007**
OTU 43	8.90	28.14	0	0	0	0	0	0								
OTU 34	0.02	0.05	0	0	0.85	0.79	1.54	1.09	0.148	0.294	**0.068**	**0.173**	**0.001**	**0.007**	**0.001**	**0.007**
OTU 24	0	0	0.05	0.07	0.15	0.13	0.12	0.11			0.674	0.877			**0.082**	**0.187**

a *q-value: The False Discovery Rate (FDR) adjusted p-value using Benjamini and Hochberg method and the q < 0.25 after FDR correction considered significant. Statistically significant values are formatted in bold*.

**Figure 2:**
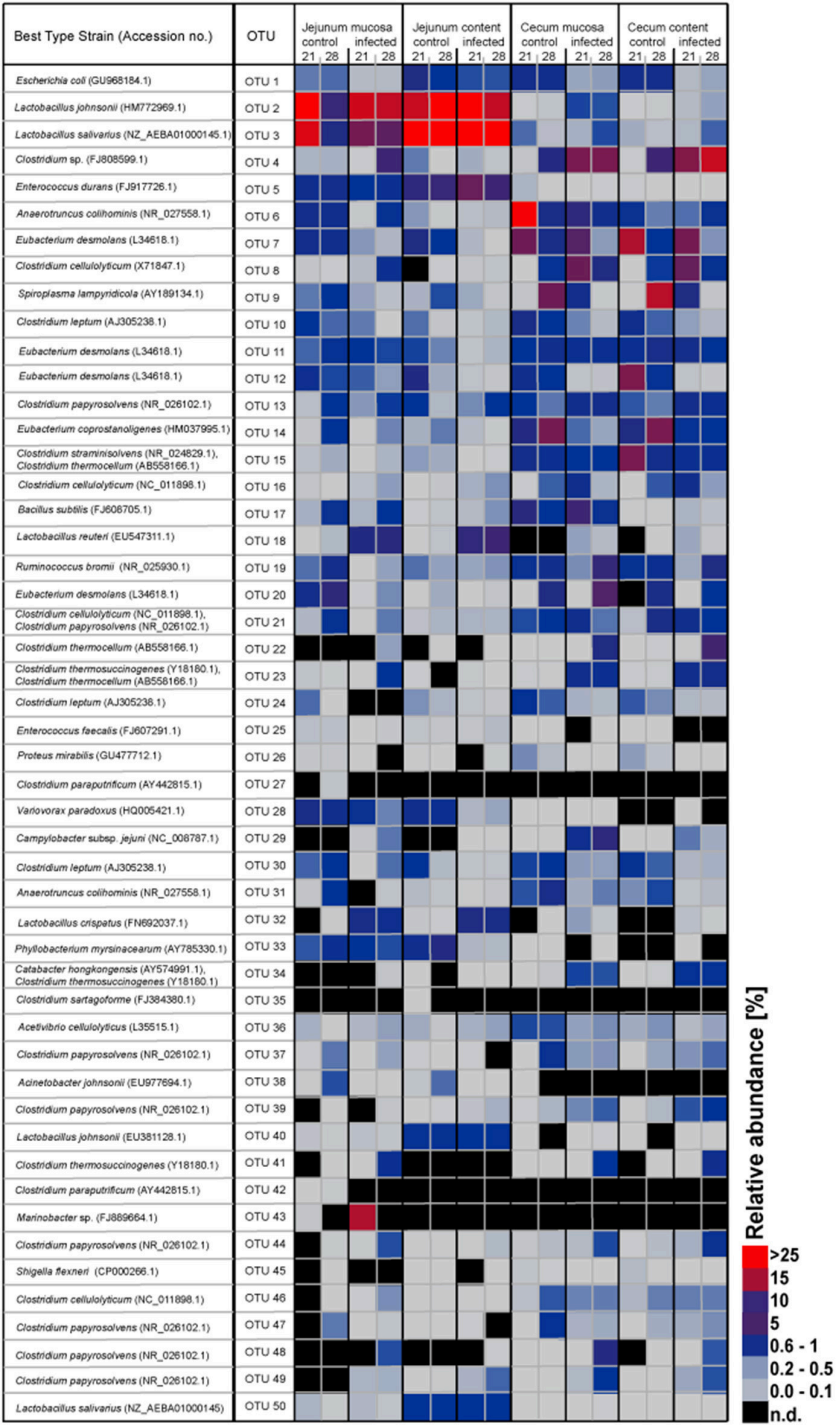
**Heatmap showing the relative abundances (%) of the 50 most-abundant OTUs sorted by gut sites of the infected birds compared with the controls at the two sampling points post infection**. The heat map integrates relative abundance of a given phylotype. Colour scaling is ranged from 0 to ≥ 25%. n.d, not detected.

**Figure 3:**
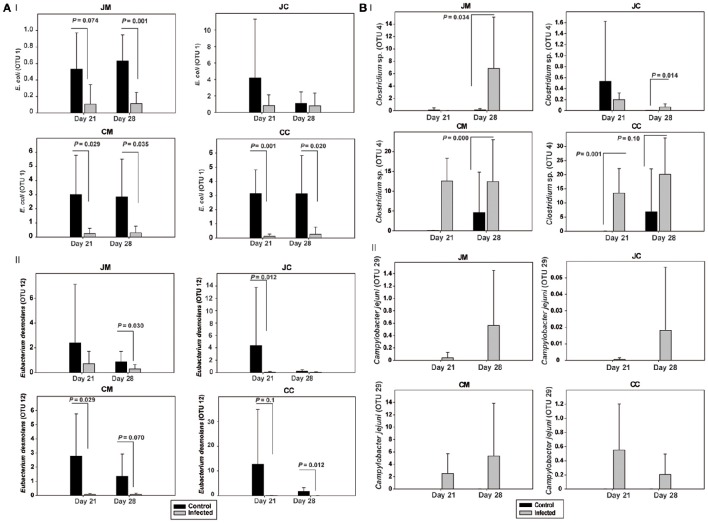
**Relative abundances (%) of the most relevant OTUs in the infected birds compared with the controls at the two sampling points post infection (A)** OTUs 1 and 12, **(B)** OTUs 4 and 29. JM, jejunal mucosa; JC, jejunal content; CM, cecum mucosa; CC, cecum content.

The OTUs and species abundances sorted by gut sites of the infected birds compared with the control birds are shown in the heatmaps (Figure 2). Interestingly, in the infected birds, the abundance of *E. coli* and *Eubacterium desmolans* (best type strain hits) were lower at different gut sites (Figure 3A). On the contrary, *Clostridium* spp. abundance was higher in the infected birds compared with the negative controls (Figure 3B).

### Assessment of the microbial community diversity

**Figure 4:**
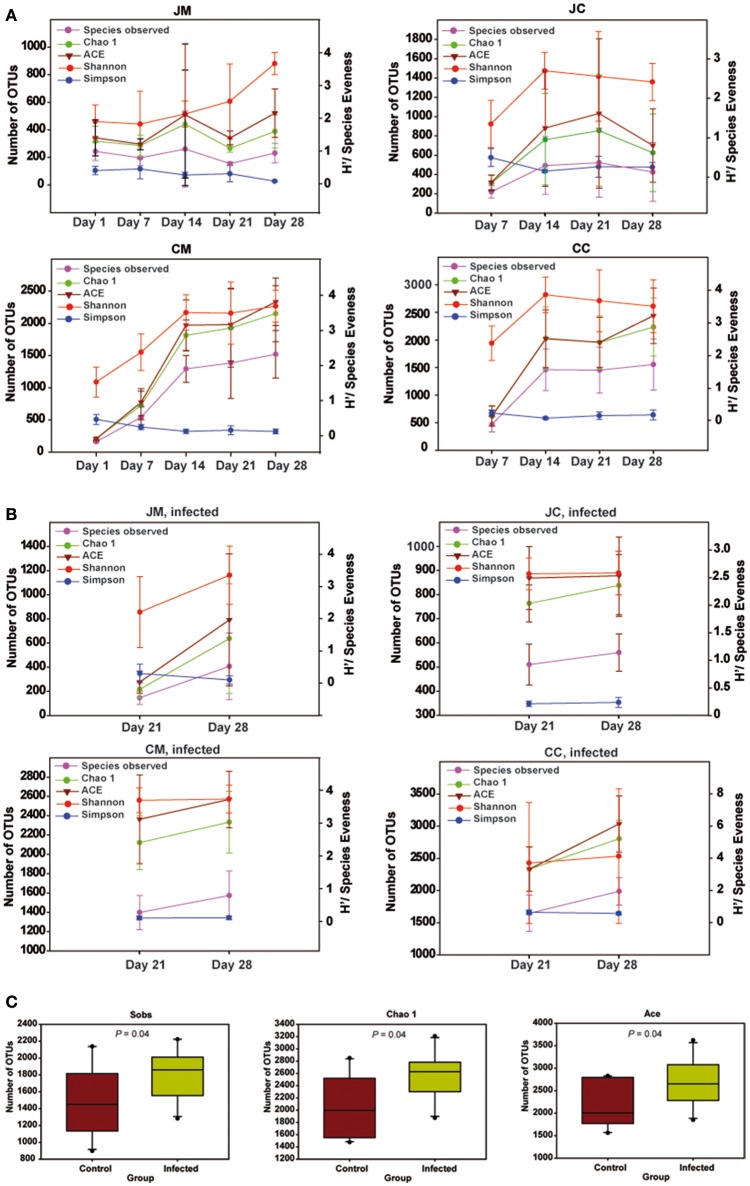
**Species richness and diversity measures of the microbial community at all gut sites in the control (A)**, infected birds **(B)**, and **(C)** species richness and diversity estimates for bacteria from cecum content of the infected birds compared with the controls. Left Y-axis for number of observed OTUs (Sobs), Chao 1 and ACE, and Right Y-axis for Shannon and Simpson. Significant differences were calculated with Kruskal-Wallis-tests and Mann-Whitney-tests, and significance was declared at *P* < 0.05. Data are presented as the mean values and *SD*. JM, jejunal mucosa; JC, jejunal content; CM, cecum mucosa; CC, cecum content.

Diversity indices estimating species richness and evenness for birds are shown in Figure 4. Diversity indices indicated that microbial richness and diversity increased with age. Interestingly, diversity indices were not different comparing samples from days 1 and 7. However, older chickens (14–28 days of age) had a significantly more diverse microbial community structure as indicated by the number of OTUs observed (Sobs), Chao1, ACE, Shannon's index, and Simpson index (*P* < 0.01). In addition, the microbial diversity in older chickens is more consistent, as there was no difference in diversity indices comparing samples from days 14 to 28. The results also revealed significant differences in the microbial diversity among jejunum and cecum as the chicken aged, supported by Sobs (*P* < 0.001), Chao1 (*P* < 0.001), ACE (*P* < 0.001), Shannon's index (*P* < 0.001), and Simpson index (*P* = 0.060) with a more complex diversity in the cecum compared with the jejunum. Furthermore, a difference in species richness among the luminal and mucosa-associated gut microbiota, independent of the age, was detected in all birds as supported by Sobs (*P* = 0.017), Chao1 (*P* = 0.015), ACE (*P* = 0.022), respectively.

In the infected birds, significant differences in the microbial diversity among jejunum and cecum supported by Sobs (*P* < 0.001), Chao1 (*P* < 0.001), ACE (*P* < 0.001), Shannon's index (*P* < 0.001), and Simpson index (*P* = 0.011) were found. Additionally, an increase in the species richness among luminal and mucosa-associated gut microbiota of the infected birds at 14 dpi compared with those from 7 dpi was obtained. Diversity indices were not significantly different among the gut sites of infected and control birds. Exceptional to this, a higher species richness was noticed in the cecum content of infected birds at 14 dpi, supported by Sobs, Chao1, and ACE (*P* = 0.047, Figure 4C), indicating that the *Campylobacter* infection increased the microbiota complexity.

### Similarity and stability of the gut microbiota composition over time

**Figure 5:**
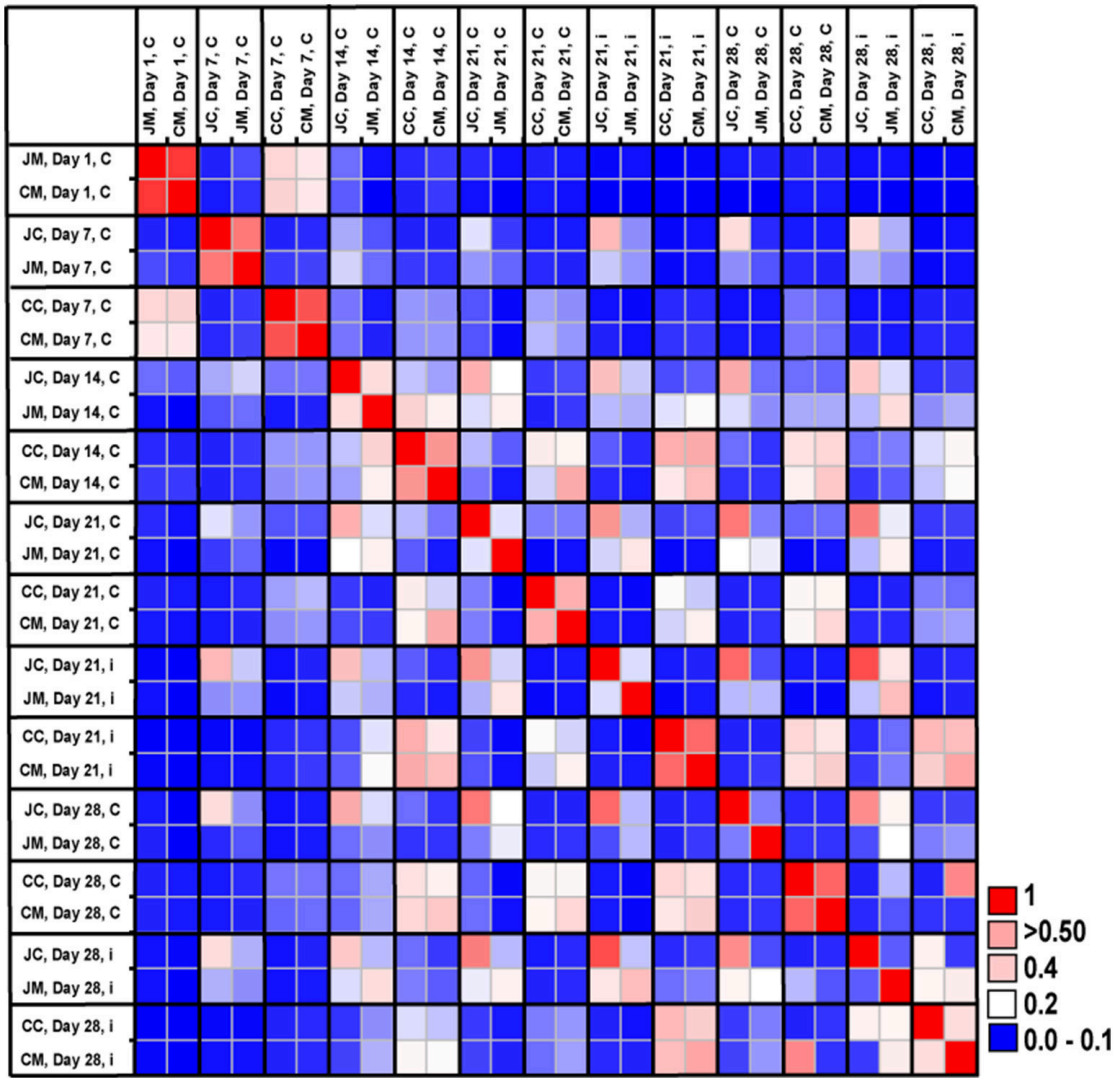
**Microbial community similarity between all samples calculated with Bray-Curtis similarities, which displays the similarity results between the control and infected groups according to age and gut sites**. JM, jejunal mucosa; JC, jejunal content; CM, cecum mucosa; CC, cecum content.

The microbial community similarity among all samples over time was assessed by calculating a Bray-Curtis similarity matrix. Community similarity analysis based on the Bray-Curtis index showed clear differences between gut sites and age, indicating strong shifts in microbial community structures (Figure 5). In addition, the Bray-Curtis index suggested that the birds at the first day of age displayed a high degree of dissimilarity compared with the other ages. It was also apparent that microbiota compositions of older birds were more similar compared with young birds.

The Bray-Curtis index revealed clear differences between jejunum and cecum from infected birds at the two sampling time points post infection. Furthermore, the comparison of the microbiota between control and infected birds showed that community structures were more dissimilar at the OTUs level, demonstrating that the gut microbial communities changed as a result of infection.

**Figure 6:**
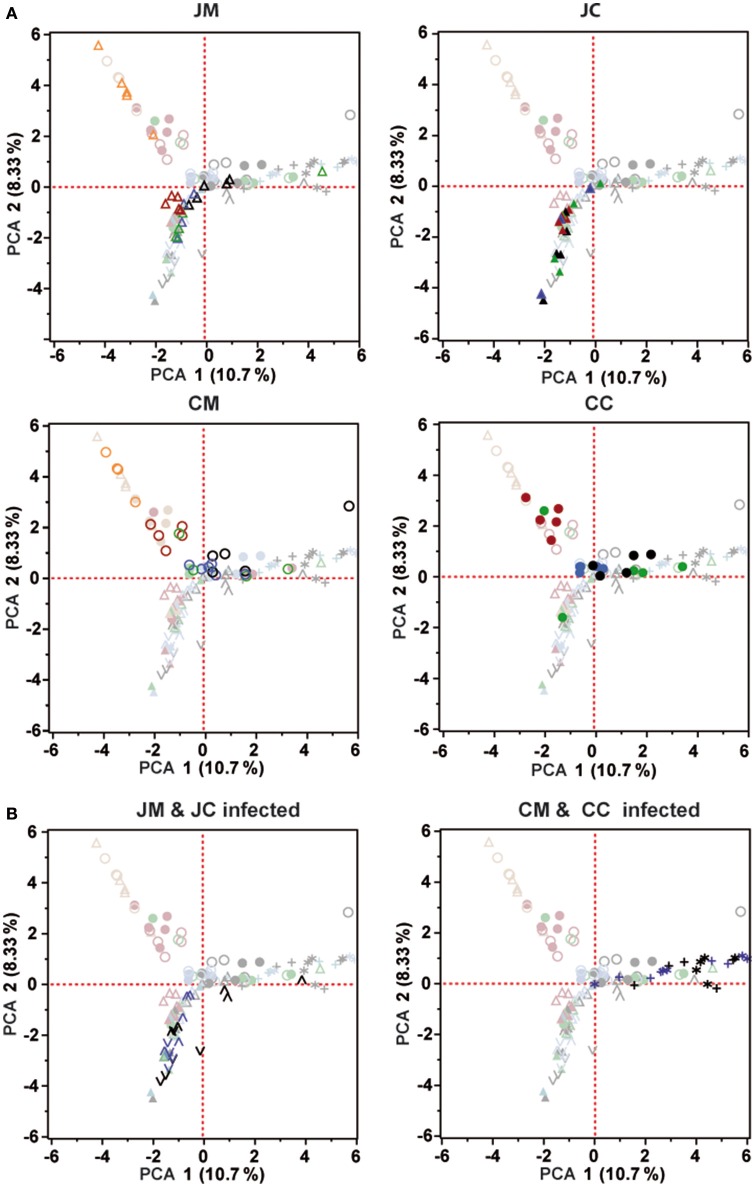
**Principal component analysis (PCA) was analyzed for the control (A)** and infected birds **(B)**. Orange (day 1), Red (day 7), Green (day 14), Blue (day 21), and Black (day 28). Each symbol indicates an individual bird. All PCA plots include the data of all samples; symbols not belonging to the sample group indicated in the header are displayed faded. JM, jejunal mucosa; JC, jejunal content; CM, cecum mucosa; CC, cecum content.

To measure the similarity between microbial communities in all birds at different ages, principal component analysis (PCA) was performed (Figure 6). PCA analysis showed that there was a clear clustering of the birds at days 1 and 7 of age in the jejunum and cecum compared with the other days. In addition, the microbial community of the older chickens clustered with less variation compared to young birds. PCA plots also demonstrate that, the microbial community was more separated in the ceca than in the jejunum.

To delineate the shared species among the groups, a Venn diagram displaying the overlaps between gut sites at different ages and groups was performed (Figure S3). The proportions of shared OTUs appear to be low at each gut site from day 1 to day 28 of age. These shared species, however, varied from one site to another.

**Figure 7:**
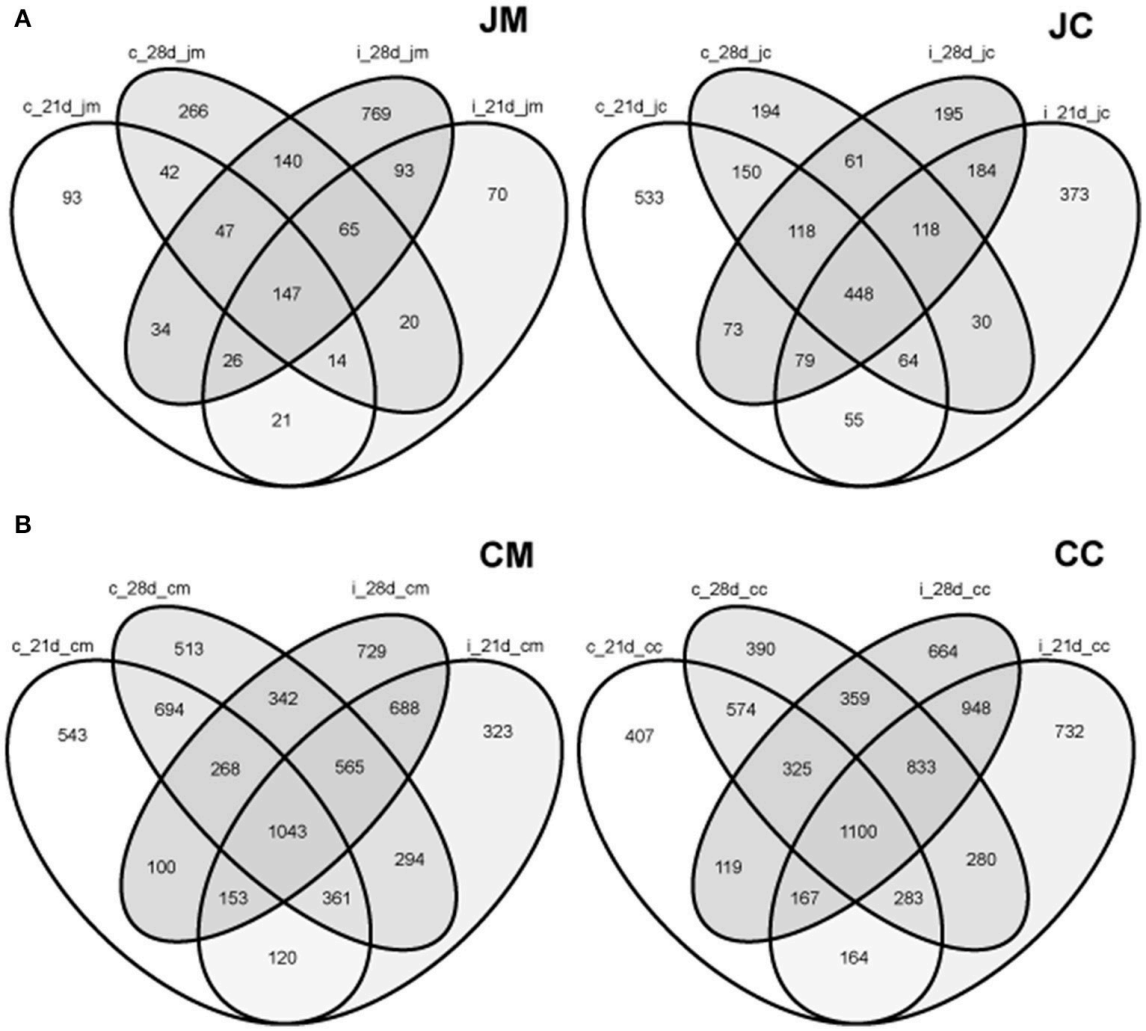
**Venn diagrams showing the shared OTUs between the control and infected birds at different gut sites at the two sampling points post infection (A)** jejunum; **(B)** cecum. JM, jejunal mucosa; JC, jejunal content; CM, cecum mucosa; CC, cecum content; control (c); infected (i); d, day.

Furthermore, the analysis showed that only 399 OTUs (*n* = 1847 OTUs) were shared among the jejunal mucosa in the control and infected birds, while 745 OTUs (*n* = 2401 OTUs) were shared between the jejunal content in the control and infected birds at the two time points post infection (Figure 7A). In the cecal mucosa and the cecal content, the comparison revealed that only 2218 OTUs (*n* = 6736 OTUs) and 2617 OTUs (*n* = 6860 OTUs) were shared, among control and infected birds combining the two time points post infection (Figure 7B). These data demonstrated that 25–36% of the observed OTUs in the jejunum and cecum were shared between the control and infected birds, respectively.

## Discussion

The intestinal microbiota acts as a physical barrier for incoming pathogens and plays an important role in the host resistance against infections by both direct interactions with pathogenic bacteria via competitive exclusion, such as occupation of attachment sites or consumption of nutrient sources, and indirectly by influencing the immune system via production of antimicrobial substances (Sekirov et al., 2010). Development of the gut microbiota in chickens occurs immediately after hatching and by getting older, this microbiome becomes very diverse until it reaches a relatively stable dynamic state (Pan and Yu, 2014).

Interactions of the intestinal microbiome with the host and certain microorganisms have profound effects on bird health, and are therefore of great importance for poultry production. Consequently, in the present study, the composition of the gut microbiota of chickens in a longitudinal study from day 1 to day 28 of age was analyzed and the differences between content and mucosa-associated gut microbiota were investigated. In order to extend the range of analyses comparisons were performed between control chickens and chickens infected at 14 days of age with *C. jejuni*.

In this study, a high diversity of phyla (15 in the jejunal and 4 in the cecal mucosal samples) was found at day 1 of life, indicating a rapid intake of environmental organisms after birth. In addition, the composition of the gut microbiota differed substantially between young and older birds, with *Proteobacteria* being significantly more present at the first day of life and decreasing thereafter, whereas the *Firmicutes* were the predominant phylum in older birds. This is in agreement with Lu et al. (2003) who found that the gut is firstly colonized by the phylum *Proteobacteria*, particularly by the family *Enterobacteriaceae*. In older birds, the phylum *Firmicutes* mainly represented by *Lachnospiraceae, Ruminococcaceae, Clostridiaceae*, and *Lactobacillaceae* dominated. As a consequence, the chicken gut is firstly colonized by facultative aerobes which are substituted later on by anaerobes. Obviously, oxygen consumption by the aerobic bacteria alters the gut ecosystem toward more reducing conditions, which facilitates subsequent growth and colonization of the obligate anaerobes (Wise and Siragusa, 2007).

Besides *Proteobacteria* and *Firmicutes*, also lower abundant phyla (e.g., *Actinobacteria and Tenericutes*) changed significantly with time, indicating high dynamics in the re-organization of the whole microbiome through time. Taken together, the present study revealed that the chicken gut is largely dominated by the phyla *Proteobacteria* and *Firmicutes*, with lower proportions of *Actinobacteria, Bacteroidetes*, and *Tenericutes*. Similarly, previous studies have also shown that *Firmicutes, Bacterioidetes*, and *Proteobacteria* are the most common phyla in the chicken ceca (Wei et al., 2013; Oakley et al., 2014; Sergeant et al., 2014).

Interestingly, jejunal, and cecal microbiota were found to be distinct and certain acid-tolerant bacteria, mostly *Acidobacteria*, were present in the jejunum only. Altogether, the results demonstrated that the abundance of bacteria varied between the jejunum and the cecum, with some species more present in the jejunum (e.g., *Acinetobacter* and *Acidobacteria*) and others (e.g., *Bacteroides* and *Clostridium*) being predominant in the cecum of chickens. This and other variations can be explained by the fact that feed passes quickly through the foregut and is retained for hours in the hindgut. In addition, the small intestine is mainly responsible for food digestion and absorption, while the large intestine, especially the cecum, is responsible for microbial fermentation, further nutrient absorption and detoxification of substances that are harmful to the host (Gong et al., 2002).

Chickens investigated in the current study had a high abundance of *E. coli* and *E. faecalis* (best type strain hits) in the first week of life which might potentially increase their resistance to other bacterial infections. *E. coli*, a facultative anaerobe bacterium, was the dominant species in the early life of chickens. Thus, a depletion of *E. coli* during the second week of life could potentially affect the host susceptibility to enteric pathogen infections, representing a key role for these gut microbiota in host resistance. This decrease in *E. coli* abundance has been attributed with a beginning dominance of anaerobes (Zhu and Joerger, 2003). It may be possible that such disturbances in the community structure allow a pathogen to colonize and proliferate. Anyhow, it remains hypothetical whether these diversity changes influence the susceptibility to pathogens and the outcome of infection.

The current results revealed that *E. coli, E. faecalis, C. paraputrificum*, and *C. sartagoforme* (best type strain hits) were more predominant in the mucosa than in the lumen, suggesting significant implications for birds' health, considering that the mucosa-associated bacteria are of great importance in the host mucosal responses with consequences for the mucosal barrier (Ott et al., 2004).

Despite the high prevalence of *Campylobacter* in chickens the mechanism of colonization in the gut is still poorly understood. The high bacterial load in the gut and the establishment of a latent infection characterized by continuous shedding indicates that *Campylobacter* in chickens can modify the microbiota composition. In the current study it could be shown that *Campylobacter* colonization shifted the two major phyla towards an enrichment of *Firmicutes* with concomitant reduction of *Proteobacteria*. Interestingly, a reverse correlation between *Firmicutes* and *Proteobacteria* was observed, suggesting a possible antagonistic interaction between these two phyla. According to Pan and Yu (2014) alterations in one phyla or species may not only affect the host directly, but can also disrupt the entire microbial community. Notably, bacterial taxa belonging to the phyla *Firmicutes* are known to be involved in the degradation of complex carbohydrates (not absorbed by the host) and in the production of SCFAs (Thibodeau et al., 2015). Thus, the SCFAs production by *Firmicutes* might, at least partially, explain their dominance in the infected birds, which have a high SCFAs requirement as a source of energy for *C. jejuni* to colonize the chicken gut. Furthermore, Brown et al. (2012) reported that members of the phylum *Firmicutes* can inhibit the growth of opportunistic pathogens, such as *E. coli*, which has also been shown in the present study.

Besides these major shifts, also low abundant phyla (e.g., *Actinobacteria* and *Tenericutes*) were affected by the *Campylobacter* infection, which could also disequilibrate the microbiome composition. Similarly, Johansen et al. (2006) found in a denaturing gradient gel electrophoresis (DGGE) based experiment that *C. jejuni* colonization affected the development and complexity of the microbial communities of the ceca over 17 days of age. Furthermore, Qu et al. (2008) noted that the community structure of the cecal microbiome from the *C. jejuni* challenged chicken has greater diversity and evenness with a higher abundance of *Firmicutes* at the expense of the *Bacteroidetes* and other taxa. Sofka et al. (2015) also reported that *Campylobacter* carriage, assessed in samples from slaughter houses, was associated with moderate modulations of the cecal microbiome as revealed by an increase in *Streptococcus* and *Blautia* relative abundance in birds of 56 days of age, originating from different farms and production types. Recently, Thibodeau et al. (2015) found also that *C. jejuni* colonization induced a moderate alteration of the chicken cecal microbiome beta-diversity at 35 days of age.

This study's results strongly suggest that the *Campylobacter* associated alterations of the gut microbiota were a direct effect due to the interaction of *C. jejuni* with the microbiota or a consequence of the host responses or even a combination of both (Barman et al., 2008; Mon et al., 2015). The obtained results indicate that the influence of a *Campylobacter* infection on microbial communities was more pronounced at 14 dpi than at 7 dpi. This could be explained by an increased load of *Camplyobacter* at the later time point as demonstrated in recent studies using the same *C. jejuni* strain (Awad et al., 2014, 2015a,b, 2016).

We also found significant differences in the abundance of certain bacterial species in the infected birds compared with the controls. *C. jejuni* caused a significant decrease in *E. coli* (best type strain hit) in the microbiota of infected birds in both jejunum and cecum. This is in agreement with our previous study which showed that *Campylobacter* colonization decreased *E. coli* loads in the jejunum and cecum at 7 dpi and at 14 dpi, but increased *E. coli* translocation to the liver and spleen of the infected birds as determined by conventional bacteriology (Awad et al., 2016). Thus, the current results pointed out that the relative abundance of *E. coli* could be an important determinant of susceptibility for a *Campylobacter* infection in particular and Gram-negative pathogens in general.

In contrast to the *Campylobacter -E. coli interaction*, it was found that the relative abundance of *Clostridium* spp. was higher in the infected birds compared with the negative controls, indicating a link between *C. jejuni* and *Clostridium*. This confirms data from an earlier study in which a positive correlation between high levels of *Clostridium perfringens* (>6 log) and the colonization of *C. jejuni* were found by real-time quantitative PCR (Skånseng et al., 2006; Thibodeau et al., 2015). This might be due to the fact that *C. jejuni* acts as a hydrogen sink leading to improved growth conditions for some *Clostridia* through increased fermentation (Kaakoush et al., 2014). This link can also be explained by the fact that the *Clostridium* organic acid production could be used by *C. jejuni* as an energy source. Furthermore, it was found that a *Campylobacter* infection induces excess mucous production in the intestine (Molnár et al., 2015) which consequently may enhance *Clostridium* proliferation due to the fact that an increase in mucin secretion in the gut provides an opportunity for *Clostridium* spp. to proliferate (M'Sadeq et al., 2015). Overall, the higher abundance of *Campylobacter* and *Clostridium* spp. might result in a higher endotoxin production with subsequent increase in intestinal permeability that facilitates the colonization and enhances bacterial translocation from the intestine to the internal organs, which is well in agreement with our pervious results (Awad et al., 2015a, 2016).

Finally, the strong shifts in the bacterial microbiome in the current study might help to explain why a *Campylobacter* infection is age dependent and chickens in the field become mainly colonized at an age of two to 4 weeks (Newell and Fearnley, 2003; Conlan et al., 2007). In agreement with this, Bereswill et al. (2011) demonstrated that a shift of intestinal microbiota in humans was linked with an increased susceptibility for *C. jejuni*. Finally, Haag et al. (2012) demonstrated that *C. jejuni* colonization in mice depends on the microbiota of the host and *vice versa* and *Campylobacter* colonization induces a shift of the intestinal microbiota. This was also observed in the present study as community structures were more dissimilar at the OTUs level in the infected birds compared with the controls. Moreover, in the infected birds, the population of beneficial microbes, such as *E. coli* and *E. desmolans* were comparatively lower than the potentially pathogenic bacteria, such as *Clostridium* spp., rendering the need for modulation of the gut microbiota to improve the gut health of the infected birds.

## Conclusion

In the current study a substantial change in the composition of luminal and mucosa-associated gut microbiota in broiler chickens from day 1–28 was noticed. It could also be demonstrated that a *C. jejuni* infection in chickens was associated with significant changes in the composition of the intestinal ecosystem. Furthermore, these changes of the gut microbiota could lead to intestinal dysfunction, which has been evidenced in our previous studies. In this context, the results provide new insights into the microecological divergence of the intestinal microbiota with and without a *Campylobacter* infection and illustrate the *C. jejuni*–host crosstalk within the gut of broiler chickens. Understanding the relationship between disruption of the normal gut microbiota and *Campylobacter* infection may lead to improve in control strategies in order to minimize the consequences for the chicken host and the risk of bacterial spread to humans.

## Author contributions

All authors listed, have made substantial, direct and intellectual contribution to the work, and approved it for publication.

### Conflict of interest statement

The authors declare that the research was conducted in the absence of any commercial or financial relationships that could be construed as a potential conflict of interest.

## Supplementary material

Figure S1 **Relative abundances (%) of the most abundant bacterial phyla of (A)** control and **(B)** infected birds. Data are presented as the mean values and SD. Left Y-axis for *Firmicutes, Proteobacteria* and others (unclassified); Right Y-axis for *Tenericutes, Acidobacteria, Actinobacteria, Bacteroides*, and *Cyanobacteria*. JM, jejunal mucosa; JC, jejunal content; CM, cecum mucosa; CC, cecum content.

Figure S2 **Heatmap showing the relative abundances of the 50 most-abundant OTUs sorted by gut sites and age in the control birds**. The heat map shows relative abundance of a given phylotype. Colour scaling is ranged from 0 to higher than 25%. n.d, not detected; n.a, not analyzed.

Figure S3 **Venn diagrams are showing the shared OTUs for the control at different gut sites from day 1–28 (A)** jejunum; **(B)** cecum. jm, jejunal mucosa; jc, jejunal content; cm, cecum mucosa; cc, cecum content; (c), control; (i), infected; d, day.

Table S1 **Relative abundances (%) of bacterial phyla in the control birds from day 1–28 (A)** jejunal mucosa, **(B)** jejunal content, **(C)** cecal mucosa, **(D)** cecal content.

Table S2 **Relative abundances (%) of bacterial phyla in different gut sites of control birds (day 1–28)**.

Table S3 **Relative abundances (%) of bacterial phyla in different gut sites of infected birds (days 21 and 28)**.

Table S4 **Relative abundances (%) of bacterial phyla in the infected birds at the two sampling points post infection (A)** jejunal mucosa, **(B)** jejunal content, **(C)** cecal mucosa, **(D)** cecal content.

Table S5 **The 50 most abundant OTUs in the control birds from day 1–28 (A)** jejunal mucosa, **(B)** jejunal content, **(C)** cecal mucosa, **(D)** cecal content.

Table S6 **The 50 most abundant OTUs in the infected birds at the two sampling points post infection (A)** jejunal mucosa, **(B)** jejunal content, **(C)** cecal mucosa, and **(D)** cecal content.
