# Supplementary figures and images for: Age-Related Differences in the Luminal and Mucosa-Associated Gut Microbiome of Broiler Chickens and Shifts Associated with Campylobacter jejuni Infection
Source: Front Cell Infect Microbiol. 2016 Nov 22;6:154. doi: 10.3389/fcimb.2016.00154 (PMC5118433; doi:10.3389/fcimb.2016.00154)

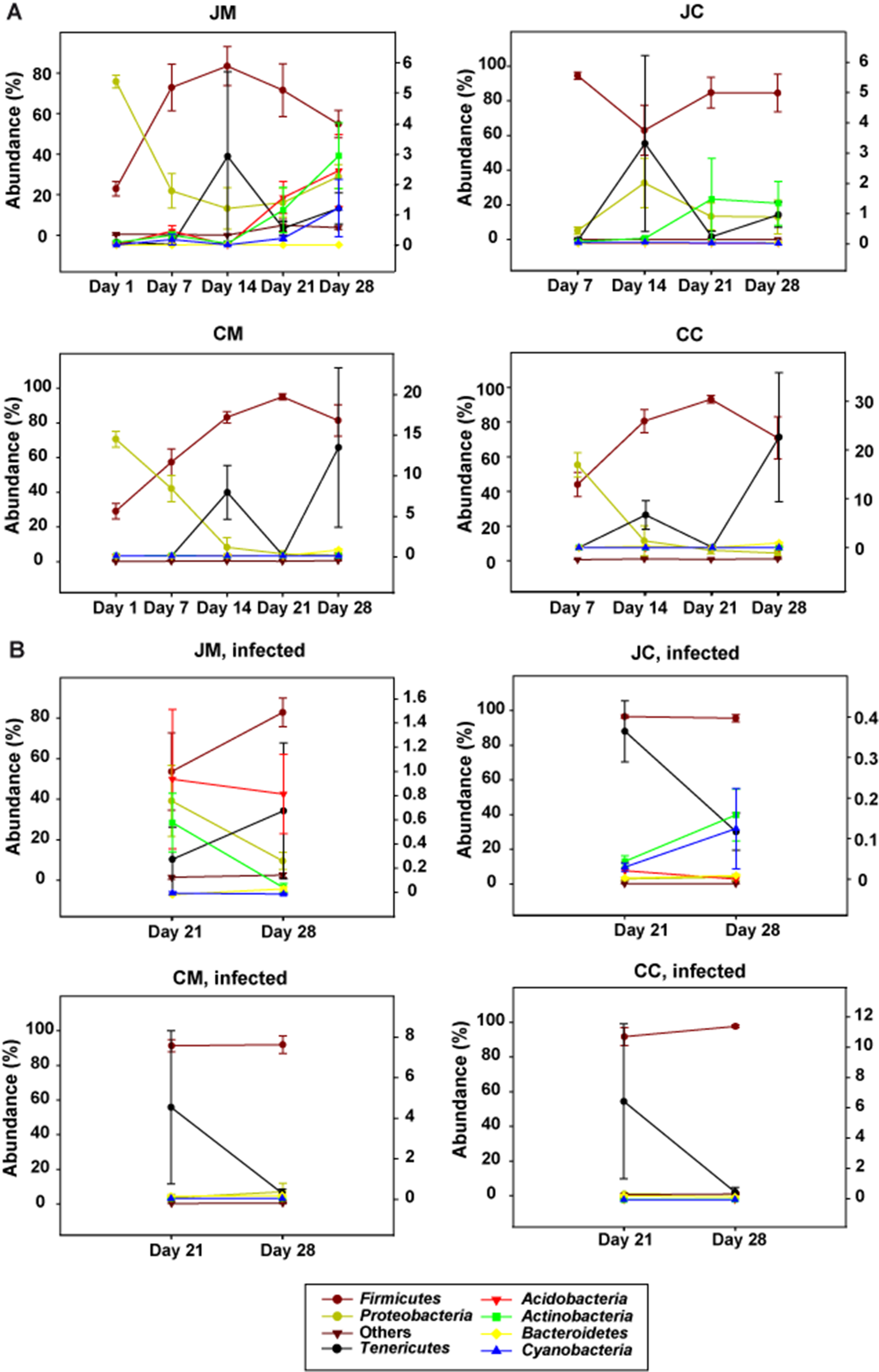

Supplement: Figure S1 — Relative abundances (%) of the most abundant bacterial phyla of (A) control and (B) infected birds. Data are presented as the mean values and SD. Left Y-axis for Firmicutes, Proteobacteria and others (unclassified); Right Y-axis for Tenericutes, Acidobacteria, Actinobacteria, Bacteroides, and Cyanobacteria. JM, jejunal mucosa; JC, jejunal content; CM, cecum mucosa; CC, cecum content. [file Image1.TIF]

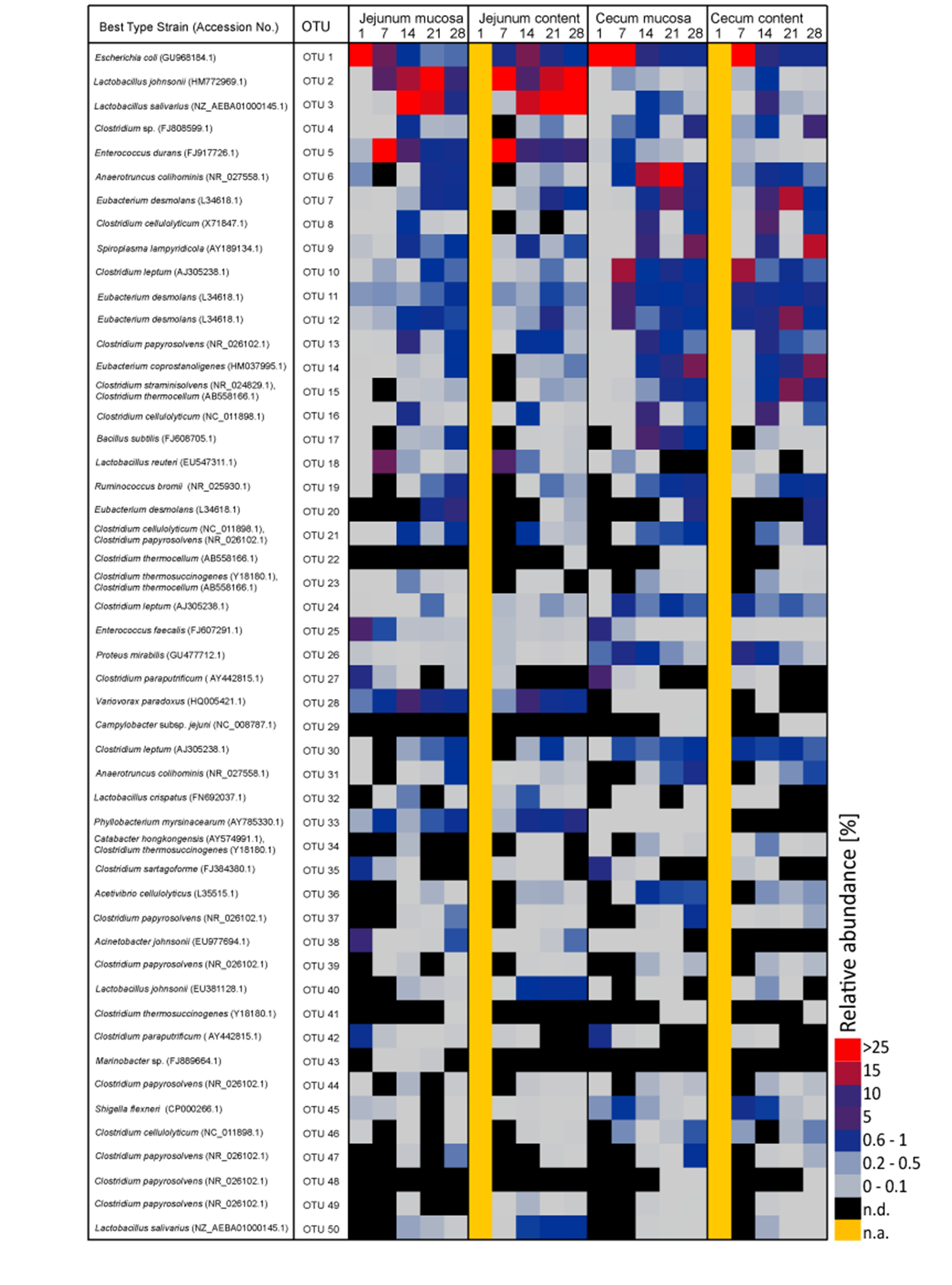

Supplement: Figure S2 — Heatmap showing the relative abundances of the 50 most-abundant OTUs sorted by gut sites and age in the control birds. The heat map shows relative abundance of a given phylotype. Colour scaling is ranged from 0 to higher than 25%. n.d, not detected; n.a, not analyzed. [file Image2.TIF]

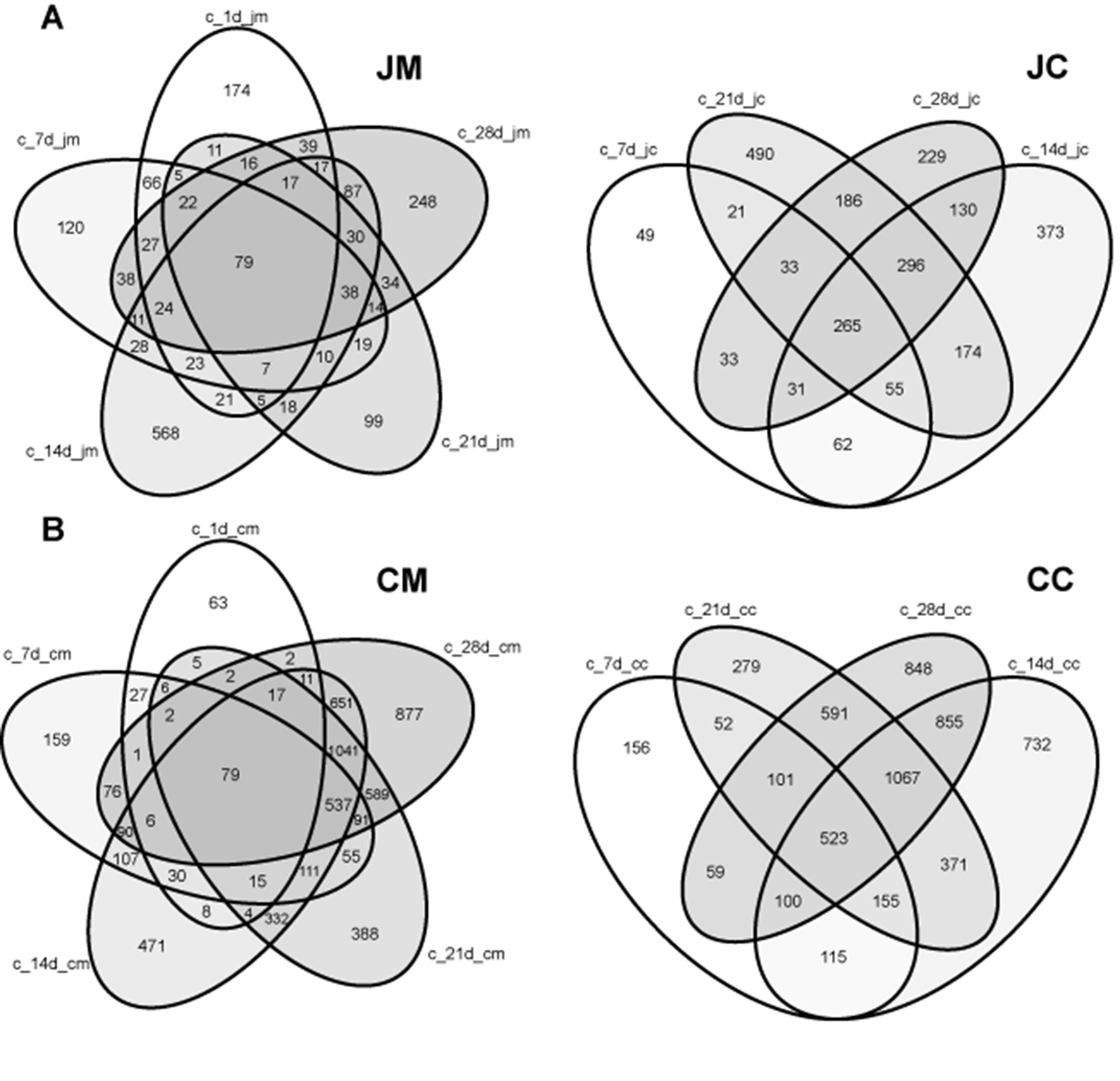

Supplement: Figure S3 — Venn diagrams are showing the shared OTUs for the control at different gut sites from day 1–28 (A) jejunum; (B) cecum. jm, jejunal mucosa; jc, jejunal content; cm, cecum mucosa; cc, cecum content; (c), control; (i), infected; d, day. [file Image3.TIF]
