# Supplementary material for: Age-Related Differences in the Luminal and Mucosa-Associated Gut Microbiome of Broiler Chickens and Shifts Associated with Campylobacter jejuni Infection
Source: Front Cell Infect Microbiol. 2016 Nov 22;6:154. doi: 10.3389/fcimb.2016.00154 (PMC5118433; doi:10.3389/fcimb.2016.00154)
Supplement: Table S2 — Relative abundances (%) of bacterial phyla in different gut sites of control birds (day 1–28). [file Table2.DOC]

**TABLE S2 |** Relative abundances (%) of bacterial phyla in different gut sites of control birds (day 1- 28).

| **Phylum** | **Jejunum mucosa** | |  | **Jejunum content** | |  | **Cecum mucosa** | |  | **Cecum content** | |  | **JM-JC** | | **CM-CC** | | **JM-CM** | | **JC-CC** | |
| --- | --- | --- | --- | --- | --- | --- | --- | --- | --- | --- | --- | --- | --- | --- | --- | --- | --- | --- | --- | --- |
|  | **Mean** | **SD** |  | **Mean** | **SD** |  | **Mean** | **SD** |  | **Mean** | **SD** |  | ***P* values** | **q values^1^** | ***P* values** | **q values^1^** | ***P* values** | **q values^1^** | ***P* values** | **q values^1^** |
| ***Parvarchaeota*** | 0.05 | 0.04 |  | 0 | 0 |  | 0 | 0 |  | 0 | 0 |  |  |  |  |  |  |  |  |  |
| **AC1** | 0.02 | 0.01 |  | 0 | 0 |  | 0 | 0 |  | 0 | 0 |  |  |  |  |  |  |  |  |  |
| ***Acidobacteria*** | 0.87 | 0.32 |  | 0 | 0 |  | 0 | 0 |  | 0 | 0 |  |  |  |  |  |  |  |  |  |
| ***Actinobacteria*** | 0.90 | 0.33 |  | 0.74 | 0.38 |  | 0.02 | 0.01 |  | 0.01 | 0 |  | 0.801 | 0.853 | 0.653 | 0.809 | **0.001** | **0.004** | **0.001** | **0.004** |
| ***Armatimonadetes*** | 0.15 | 0.15 |  | 0 | 0 |  | 0 | 0 |  | 0 | 0 |  |  |  |  |  |  |  |  |  |
| ***Bacteroidetes*** | 0.01 | 0 |  | 0.01 | 0 |  | 0.16 | 0.07 |  | 0.30 | 0.10 |  | 0.719 | 0.818 | 0.271 | 0.419 | **0.007** | **0.023** | **0.003** | **0.011** |
| ***Chloroflexi*** | 0.43 | 0.28 |  | 0 | 0 |  | 0 | 0 |  | 0 | 0 |  |  |  |  |  | **0.041** | **0.090** |  |  |
| ***Crenarchaeota*** | 0.14 | 0.11 |  | 0 | 0 |  | 0 | 0 |  | 0 | 0 |  |  |  |  |  |  |  |  |  |
| ***Cyanobacteria*** | 0.33 | 0.21 |  | 0.01 | 0 |  | 0 | 0 |  | 0 | 0 |  | **0.030** | **0.071** | 1.000 | 1.000 | **0.001** | **0.004** | **0.001** | **0.004** |
| ***Elusimicrobia*** | 0.69 | 0.35 |  | 0 | 0 |  | 0 | 0 |  | 0 | 0 |  | **0.001** | **0.004** |  |  |  |  |  |  |
| ***Firmicutes*** | 60.72 | 5.83 |  | 81.74 | 5.33 |  | 69.29 | 5.37 |  | 72.14 | 5.48 |  | **0.014** | **0.038** | 0.686 | 0.809 | 0.279 | 0.419 | 0.216 | 0.375 |
| **GN02** | 0.16 | 0.09 |  | 0 | 0 |  | 0 | 0 |  | 0 | 0 |  |  |  |  |  |  |  |  |  |
| **NC10** | 0 | 0 |  | 0 | 0 |  | 0 | 0 |  | 0 | 0 |  |  |  |  |  |  |  |  |  |
| ***Nitrospirae*** | 0.24 | 0.10 |  | 0 | 0 |  | 0 | 0 |  | 0 | 0 |  |  |  |  |  |  |  |  |  |
| **OD1** | 0.22 | 0.11 |  | 0 | 0 |  | 0 | 0 |  | 0 | 0 |  |  |  |  |  | **0.016** | **0.041** |  |  |
| **OP3** | 0 | 0 |  | 0 | 0 |  | 0 | 0 |  | 0 | 0 |  |  |  |  |  |  |  |  |  |
| ***Planctomycetes*** | 0.13 | 0.05 |  | 0 | 0 |  | 0 | 0 |  | 0 | 0 |  |  |  |  |  |  |  |  |  |
| ***Proteobacteria*** | 31.83 | 5.71 |  | 16.10 | 4.95 |  | 25.91 | 5.77 |  | 19.31 | 5.48 |  | **0.049** | **0.101** | 0.387 | 0.555 | 0.478 | 0.657 | 0.668 | 0.809 |
| ***Tenericutes*** | 0.98 | 0.59 |  | 1.13 | 0.74 |  | 4.26 | 2.20 |  | 7.33 | 3.75 |  | 0.761 | 0.837 | 0.518 | 0.684 | 0.141 | 0.258 | **0.073** | **0.142** |
| **TM7** | 0.19 | 0.11 |  | 0.16 | 0.11 |  | 0 | 0 |  | 0 | 0 |  | 0.886 | 0.914 |  |  |  |  | 0.260 | 0.419 |
| ***Verrucomicrobia*** | 0.04 | 0.03 |  | 0 | 0 |  | 0 | 0 |  | 0 | 0 |  |  |  |  |  |  |  |  |  |
| **WPS-2** | 0.01 | 0.01 |  | 0 | 0 |  | 0 | 0 |  | 0 | 0 |  |  |  |  |  |  |  |  |  |
| **ZB3** | 0.09 | 0.09 |  | 0 | 0 |  | 0 | 0 |  | 0 | 0 |  |  |  |  |  |  |  |  |  |
| **Others** | 1.80 | 0.62 |  | 0.10 | 0.02 |  | 0.36 | 0.05 |  | 0.90 | 0.12 |  | **0.001** | **0.004** | **0.001** | **0.004** | **0.009** | **0.027** | **0.001** | **0.004** |

^1^ q-value: the False Discovery Rate (FDR) adjusted p-value using Benjamini and Hochberg method and the q values < 0.25 after FDR correction considered significant.
