# Supplementary material for: Age-Related Differences in the Luminal and Mucosa-Associated Gut Microbiome of Broiler Chickens and Shifts Associated with Campylobacter jejuni Infection
Source: Front Cell Infect Microbiol. 2016 Nov 22;6:154. doi: 10.3389/fcimb.2016.00154 (PMC5118433; doi:10.3389/fcimb.2016.00154)
Supplement: Table S3 — Relative abundances (%) of bacterial phyla in different gut sites of infected birds (days 21 and 28). [file Table3.DOCX]

**TABLE S3 |** Relative abundances (%) of bacterial phyla in different gut sites of infected birds (days 21 and 28).

| **Phylum** | **Jejunum mucosa** | |  | **Jejunum content** | |  | **Cecum mucosa** | |  | **Cecum content** | |  | **JM-JC** | | **CM-CC** | | **JM-CM** | | **JC-CC** | |
| --- | --- | --- | --- | --- | --- | --- | --- | --- | --- | --- | --- | --- | --- | --- | --- | --- | --- | --- | --- | --- |
|  | **Mean** | **SD** |  | **Mean** | **SD** |  | **Mean** | **SD** |  | **Mean** | **SD** |  | ***P* values** | **q values^1^** | ***P* values** | **q values^1^** | ***P* values** | **q values^1^** | ***P* values** | **q values^1^** |
| ***Parvarchaeota*** | 0.01 | 0.01 |  | 0 | 0 |  | 0 | 0 |  | 0 | 0 |  |  |  |  |  |  |  |  |  |
| **AC1** | 0 | 0 |  | 0 | 0 |  | 0 | 0 |  | 0 | 0 |  |  |  |  |  |  |  |  |  |
| ***Acidobacteria*** | 0.89 | 0.31 |  | 0.01 | 0.01 |  | 0 | 0 |  | 0 | 0 |  | **0.002** | **0.010** |  |  | **0.002** | **0.010** |  |  |
| ***Actinobacteria*** | 0.33 | 0.15 |  | 0.10 | 0.04 |  | 0 | 0 |  | 0 | 0 |  | 0.167 | **0.220** | **0.008** | **0.019** | **0.001** | **0.007** | **0.001** | **0.007** |
| ***Armatimonadetes*** | 0.04 | 0.04 |  | 0 | 0 |  | 0 | 0 |  | 0 | 0 |  |  |  |  |  |  |  |  |  |
| ***Bacteroidetes*** | 0.02 | 0.01 |  | 0.01 | 0 |  | 0.12 | 0.03 |  | 0.22 | 0.05 |  | 0.191 | **0.241** | 0.134 | **0.185** | **0.007** | **0.018** | **0.001** | **0.007** |
| ***Chloroflexi*** | 0.24 | 0.20 |  | 0 | 0 |  | 0 | 0 |  | 0 | 0 |  |  |  |  |  |  |  |  |  |
| ***Crenarchaeota*** | 0.02 | 0.01 |  | 0 | 0 |  | 0 | 0 |  | 0 | 0 |  |  |  |  |  |  |  |  |  |
| ***Cyanobacteria*** | 0.01 | 0.01 |  | 0.08 | 0.05 |  | 0 | 0 |  | 0 | 0 |  | **0.071** | **0.103** |  |  |  |  |  |  |
| ***Elusimicrobia*** | 1.17 | 0.65 |  | 0 | 0 |  | 0 | 0 |  | 0 | 0 |  |  |  |  |  |  |  |  |  |
| ***Firmicutes*** | 68.24 | 10.74 |  | 96.08 | 1.08 |  | 91.66 | 2.89 |  | 94.69 | 2.66 |  | **0.003** | **0.012** | 0.432 | 0.501 | **0.027** | **0.052** | 0.808 | 0.808 |
| **GN02** | 0.79 | 0.70 |  | 0 | 0 |  | 0 | 0 |  | 0 | 0 |  |  |  |  |  |  |  |  |  |
| **NC10** | 0 | 0 |  | 0 | 0 |  | 0 | 0 |  | 0 | 0 |  |  |  |  |  |  |  |  |  |
| ***Nitrospirae*** | 0.51 | 0.37 |  | 0 | 0 |  | 0 | 0 |  | 0 | 0 |  |  |  |  |  |  |  |  |  |
| **OD1** | 0.20 | 0.10 |  | 0 | 0 |  | 0 | 0 |  | 0 | 0 |  |  |  |  |  |  |  |  |  |
| **OP3** | 0.09 | 0.09 |  | 0 | 0 |  | 0 | 0 |  | 0 | 0 |  |  |  |  |  |  |  |  |  |
| ***Planctomycetes*** | 0.19 | 0.13 |  | 0 | 0 |  | 0 | 0 |  | 0 | 0 |  |  |  |  |  |  |  |  |  |
| ***Proteobacteria*** | 24.39 | 9.81 |  | 3.39 | 1.01 |  | 5.39 | 2.55 |  | 0.86 | 0.24 |  | **0.006** | **0.017** | **0.060** | **0.092** | **0.052** | **0.084** | **0.005** | **0.016** |
| ***Tenericutes*** | 0.49 | 0.30 |  | 0.24 | 0.06 |  | 2.37 | 1.92 |  | 3.50 | 2.61 |  | 0.508 | 0.567 | 0.565 | 0.585 | 0.540 | 0.580 | **0.041** | **0.070** |
| **TM7** | 0.26 | 0.18 |  | 0 | 0 |  | 0 | 0 |  | 0 | 0 |  | **0.024** | **0.050** |  |  | **0.024** | **0.050** |  |  |
| ***Verrucomicrobia*** | 0.01 | 0 |  | 0 | 0 |  | 0 | 0 |  | 0 | 0 |  |  |  |  |  |  |  |  |  |
| **WPS-2** | 0.03 | 0.03 |  | 0 | 0 |  | 0 | 0 |  | 0 | 0 |  |  |  |  |  |  |  |  |  |
| **ZB3** | 0 | 0 |  | 0 | 0 |  | 0 | 0 |  | 0 | 0 |  |  |  |  |  |  |  |  |  |
| **Others** | 2.07 | 0.69 |  | 0.09 | 0.01 |  | 0.45 | 0.11 |  | 0.74 | 0.24 |  | **0.005** | **0.016** | 0.302 | 0.365 | **0.036** | **0.065** | **0.001** | **0.007** |

^1^ q-value: the False Discovery Rate (FDR) adjusted p-value using Benjamini and Hochberg method and the q values < 0.25 after FDR correction considered significant.
